# Supplementary material for: Accuracy of rapid lateral flow immunoassays for human leptospirosis diagnosis: A systematic review and meta-analysis
Source: PLoS Negl Trop Dis. 2024 May 15;18(5):e0012174. doi: 10.1371/journal.pntd.0012174 (PMC11132494; doi:10.1371/journal.pntd.0012174)
Supplement: S1 Table — (DOCX) [file pntd.0012174.s003.docx]

**S1 Table** Criteria for assessing risk of bias of studies included in this review

| **Category** | **Criteria** |
| --- | --- |
| Patient selection | 1. Cases and controls selected from the same population 2. Eligibility determined by the presence of possible leptospirosis |
| Index Test | 1. Assessors blinded to results of reference test when performing LFA test 2. Threshold for positivity is defined a priori and in keeping with manufacturers' recommendations |
| Reference Test | 1. Cases defined as leptospirosis with a four-fold rise in antibody titers on MAT or IFA, or positive culture, or detection of leptospira DNA |
| Flow and timing | 1. All patients subjected to the same reference tests 2. Reference tests and index tests performed on samples taken at the same time for illness |
