# Supplementary material for: Accuracy of rapid lateral flow immunoassays for human leptospirosis diagnosis: A systematic review and meta-analysis
Source: PLoS Negl Trop Dis. 2024 May 15;18(5):e0012174. doi: 10.1371/journal.pntd.0012174 (PMC11132494; doi:10.1371/journal.pntd.0012174)
Supplement: S2 Table — (DOCX) [file pntd.0012174.s004.docx]

**S2 Table** Criteria for assessing the applicability of the studies included in this review

| **Category** | **Criteria** |
| --- | --- |
| Patient selection | Patients included with febrile illness and with a duration of clinical illness from 1-21 days |
| Index Test | Test used and interpreted according to manufacturer instructions |
| Reference Test | Reference test included MAT with a panel of antigens representing likely circulating serovars |
