# Supplementary material for: Accuracy of rapid lateral flow immunoassays for human leptospirosis diagnosis: A systematic review and meta-analysis
Source: PLoS Negl Trop Dis. 2024 May 15;18(5):e0012174. doi: 10.1371/journal.pntd.0012174 (PMC11132494; doi:10.1371/journal.pntd.0012174)
Supplement: S3 Table — (DOCX) [file pntd.0012174.s005.docx]

**S3 Table** Reasons for exclusion

| **No** | **First author, year** | **Reason(s) for exclusion** | **Ref.** |
| --- | --- | --- | --- |
| 1 | K. Sapna, 2022 | Index test is not a rapid LFI | [1] |
| 2 | Revathi Poonati, 2019 | Non-human leptospirosis | [2] |
| 3 | Sonia Arafah, 2019 | Only abstract is available | [3] |
| 4 | Hiroko Iwasaki, 2016 | Insufficient data for sensitivity and specificity calculation | [4] |
| 5 | Shanmugam Vanithamani, 2015 | Insufficient data for sensitivity and specificity calculation | [5] |
| 6 | Tanvi Panwala, 2015 | Non-human leptospirosis | [6] |
| 7 | María de los A. Valverde J, 2007 | Index test is not a rapid LFI | [7] |
| 8 | Doojdao Boonyod, 2005 | Index test is not a rapid LFI | [8] |
| 9 | Marisa Kemapunmanus, 2004 | Index test is not a rapid LFI | [9] |
| 10 | Mary D. Bajani, 2003 | Index test is not a rapid LFI | [10] |
| 11 | Paul V. Effler, 2002 | Index test is not a rapid LFI | [11] |
| 12 | Paul N. Levett, 2001 | Index test is not a rapid LFI | [12] |
| 13 | Henk L. Smits, 2001 | Index test is not a rapid LFI | [13] |
| 14 | Mohammad Hatta, 2000 | Index test is not a rapid LFI | [14] |
| 15 | Henk L. Smits, 2000 | Index test is not a rapid LFI | [15] |
| 16 | Henk L. Smits, 1999 | Index test is not a rapid LFI | [16] |
| 17 | S. C. Sehgal, 1999 | Index test is not a rapid LFI | [17] |
| 18 | Claude Yersin, 1999 | Index test is not a rapid LFI | [18] |

**References**

1. Sapna K, Ashaiba A, Kumar TNR, Shashidhar V, Arun AB, Prasad KS. Evaluation of anti-LipL32 carbon nanotube immunofluorescence probe (carbo-lip) and comparison with MAT, IgM ELISA, IgM spot test and culture for early detection of leptospirosis at local hospital. J Microbiol Methods. 2022;195: 106448. doi:10.1016/j.mimet.2022.106448

2. Poonati R, Mallepaddi PC, Punati RD, Maity SN, Alamuri A, Manchikalapudi S, et al. Development and validation of point of care diagnostics for the rapid detection of multiple species of Leptospira at resource-limited areas. Curr Trends Biotechnol Pharm. 2019;13: 270–282.

3. Arafah S, Blacksell S, Mayo M, Currie B, Mace A, Ongarello S, et al. Performance evaluation of a novel multiplexed lateral flow assay to identify common causes of fever in Asia and inform treatment decisions. Am J Trop Med Hyg. 2019. pp. 482–483. doi:10.4269/ajtmh.abstract2019

4. Iwasaki H, Chagan-Yasutan H, Leano PSA, Koizumi N, Nakajima C, Taurustiati D, et al. Combined antibody and DNA detection for early diagnosis of leptospirosis after a disaster. Diagn Microbiol Infect Dis. 2016;84: 287–291. doi:10.1016/j.diagmicrobio.2016.01.001

5. Vanithamani S, Shanmughapriya S, Narayanan R, Raja V, Kanagavel M, Sivasankari K, et al. Lipopolysaccharide specific immunochromatography based lateral flow assay for serogroup specific diagnosis of leptospirosis in India. PLoS One. 2015;10: e0137130. doi:10.1371/journal.pone.0137130

6. Panwala T, Rajdev S, Mulla S. To evaluate the different rapid screening tests for diagnosis of leptospirosis. J Clin Diagn Res. 2015;9: DC21. doi:10.7860/JCDR/2015/11188.5587

7. Valverde J M de los A, León B, Taylor L, Visona K. Development of a Lepto-IgM EIACR test to diagnose leptospirosis disease in Costa Rican patient samples. Invest Clin. 2007;48: 295–304.

8. Boonyod D, Poovorawan Y, Bhattarakosol P, Chirathaworn C. LipL32, an outer membrane protein of Leptospira, as an antigen in a dipstick assay for diagnosis of leptospirosis. Asian Pac J Allergy Immunol. 2005;23: 133.

9. Kemapunmanus M, Sretrirutchai S, Khuntikij P, Pradutkanchana S, Pradutkanchana J. A prospective evaluation of four immunodiagnotic assays for human leptospirosis. Southeast Asian J Trop Med Public Health. 2004;35: 863–867.

10. Bajani MD, Ashford DA, Bragg SL, Woods CW, Aye T, Spiegel RA, et al. Evaluation of four commercially available rapid serologic tests for diagnosis of leptospirosis. J Clin Microbiol. 2003;41: 803–809. doi:10.1128/JCM.41.2.803-809.2003

11. Effler P V., Bogard AK, Domen HY, Katz AR, Higa HY, Sasaki DM. Evaluation of eight rapid screening tests for acute leptospirosis in Hawaii. J Clin Microbiol. 2002;40: 1464–1469. doi:10.1128/JCM.40.4.1464-1469.2002

12. Levett PN, Branch SL, Whittington CU, Edwards CN, Paxton H. Two methods for rapid serological diagnosis of acute leptospirosis. Clinical Diagnostic Laboratory Immunology. 2001;8: 349–351. doi:10.1128/CDLI.8.2.349-351.2001

13. Smits HL, Eapen CK, Sugathan S, Kuriakose M, Gasem MH, Yersin C, et al. Lateral-flow assay for rapid serodiagnosis of human leptospirosis. Clin Diagn Lab Immunol. 2001;8: 166–169. doi:10.1128/CDLI.8.1.166-169.2001

14. Hatta M, Smits HL, Gussenhoven GC, Gooskens J. Introduction of a rapid dipstick assay for the detection of Leptospira-specific immunoglobulin m antibodies in the laboratory diagnosis of leptospirosis in a hospital in Makassar, Indonesia. Southeast Asian J Trop Med Public Health. 2000;31: 515–520.

15. Smits HL, Hartskeerl RA, Terpstra WJ. International multi-centre evaluation of a dipstick assay for human leptospirosis. Trop Med Int Health. 2000;5: 124–128. doi:10.1046/j.1365-3156.2000.00525.x

16. Smits HL, Ananyina Y V., Chereshsky A, Dancel L, Lai-A-Fat RFM, Chee HD, et al. International Multicenter Evaluation of the Clinical Utility of a Dipstick Assay for Detection ofLeptospira-Specific Immunoglobulin M Antibodies in Human Serum Specimens. J Clin Microbiol. 1999;37: 2904–2909. doi:10.1128/JCM.37.9.2904-2909.1999

17. Sehgal SC, Vijayachari P, Sharma S, Sugunan AP. LEPTO Dipstick: a rapid and simple method for serodiagnosis of acute leptospirosis. Trans R Soc Trop Med Hyg. 1999;93: 161–164. doi:10.1016/S0035-9203(99)90293-6

18. Yersin C, Bovet P, Smits HL, Perolat P. Field evaluation of a one-step dipstick assay for the diagnosis of human leptospirosis in the Seychelles. Trop Med Int Health. 1999;4: 38–45. doi:10.1046/J.1365-3156.1999.00352.X
