# Supplementary material for: Accuracy of rapid lateral flow immunoassays for human leptospirosis diagnosis: A systematic review and meta-analysis
Source: PLoS Negl Trop Dis. 2024 May 15;18(5):e0012174. doi: 10.1371/journal.pntd.0012174 (PMC11132494; doi:10.1371/journal.pntd.0012174)
Supplement: S4 Table — (DOCX) [file pntd.0012174.s006.docx]

**S4 Table** List of reference tests and case definitions used in the selected studies

| **First author, published year** | **MAT** | | **Culture** | **Nucleotide detection** | | **IFA/ELSIA** | **Ref.** |
| --- | --- | --- | --- | --- | --- | --- | --- |
|  | **definition** | **serovars** |  | **technique** | **target** | **definition** |  |
| Jirawannaporn, 2023 | 1. single titer ≥ 1:800 OR 2. ≥4-fold rising | At, Bl, Ba, Ca, Ce, Cy, Dj, Gr, He, Ic, Ja, Lo, Mn, Mi, Pn, Po, Py, Rn, Sa, Se, Sh, Ta, Sm | EMJH | RT-PCR | *LipL32* | - | [1] |
| Campos, 2023 | *The authors stated that MAT was used to define leptospirosis status of samples; however, the definition and serovars used are not given.* | | - | - | - | - | [2] |
| Bottieau, 2022 | 1. a ≥ 4-fold rising | no data | - | PCR | no data | [3] | [3] |
| Dinhuzen, 2021 | 1. single titer ≥ 1:400 OR 2. ≥4-fold rising | Sh, Au, Se, Lo, Gr, At, Sm | EMJH | RT-PCR | *LipL32* | - | [4] |
| Silpasakorn, 2020 | - | - | - | - | - | 1. single titer ≥ 1:400 OR 2. ≥4-fold rising   (IFA with At as an antigen) | [5] |
| Dawson, 2020 | 1. single titer ≥ 1:800 OR 2. ≥4-fold rising | no data | - | - | - | - | [6] |
| Rao, 2019 | 1. single titer ≥ 1:400 OR 2. ≥4-fold rising | At, Au, Ba, Bl, Ca, Gr, Ic, Ja, Po, Py, Ta, Se, Pa, and 6 local serovars | - | - | - | - | [7] |
| Alia, 2019 (pro) | 1. single titer ≥ 1:400 OR 2. ≥4-fold rising | Au, At, Ba, Ca, Ce, Gr, Ha, Ic, Ja, Py, Ta, Dj, Po, Pa, and 6 local serovars | - | RT-PCR | *LipL32* | - | [8] |
| Alia, 2019 (retro) | 1. single titer ≥ 1:400 |  | - | - | - | - | [8] |
| Amran, 2018 | 1. single titer ≥ 1:400 OR 2. ≥4-fold rising | Au, At, Ba, Ca, Ce, Gr, Ha, Ic, Ja, Py, Ta, Dj, Po, Pa, and 6 local serovars | - | - | - | - | [9] |
| Nabity, 2018 | 1. single titer ≥ 1:800 OR 2. ≥4-fold rising | no data | EMJH | - | - | - | [10] |
| Doungchawee, 2017 | 1. single titer ≥ 1:800 OR 2. negative to ≥ 1:100* OR 3. ≥4-fold rising | no data | ✓ | - | - | - | [11] |
| Lee, 2016 (Korea) | 1. single titer ≥ 1:80 OR 2. ≥4-fold rising | La, Ca | - | - | - | - | [12] |
| Lee, 2016 (Bulgaria) | 1. single titer ≥ 1:200 OR | Ic, Po, Br | - | - | - | - | [12] |
| Lee, 2016 (Argentina) | 1. single titer ≥ 1:800 OR 2. ≥4-fold rising | Ca, Cs, Gr, Co, Ic, Po, Py, Ta, Wo, Hr, Ba, Pa, Au, At, Cy, He, Ja, Pn, Se | - | - | - | - | [12] |
| Eugene, 2015 | 1. single titer ≥ 1:400 OR | Pa | - | - | - | - | [13] |
| Podgoršek, 2015 | 1. single titer ≥ 1:100 OR 2. negative to ≥ 1:100* | Gr, Ca, Se, Po, Cy, Co, Pa, Au, Py, Ba, Pn, Ja | EMJH | PCR | *rss* | - | [14] |
| Niloofa, 2015 | 1. single titer ≥ 1:400 OR 2. negative to ≥ 1:100* OR 3. ≥4-fold rising | Pa | - | - | - | - | [15] |
| Colt, 2014 | 1. single titer ≥ 1:400 OR 2. ≥4-fold rising | Po, Se, Ta, Gr, Ce, Co, Au, Py, Ca, He, Mi, Sa, At, Cy, Bl, Ba, Dj, Ja, Pn, Sh, Ph | - | - | - | - | [16] |
| Chang, 2014 | 1. single titer ≥ 1:400 | Pa, Au, At, Bl, Ba, Ca, Ce, He, Cy, Gr, Ic, Ja, Po, Py, Ta, Hr, Se, Dj | - | PCR | secY | - | [17] |
| Widiyanti, 2013 | 1. single titer ≥ 1:400 | Co, Pa, Pi, Ma, Py, Ca, Sm, Ls, Ra | - | PCR | *rss, flaB* | - | [18] |
| Goris, 2013 | 1. single titer ≥ 1:400 OR 2. seroconversion OR 3. ≥4-fold rising | Br, Bl, Ca, Gr, He, Ic, Co, Pi, Po, Pr, Hr, Sx, Se, Pa (additional: Au, Rc, Ba, Ce, Cy, Mi, Py, Sh, Ta, An, Sm) | EMJH | - | - | 1. single titer ≥ 1:400 OR 2. seroconversion OR 3. ≥4-fold rising   (IgG ELISA with Co as an antigen) | [19] |
| Nabity, 2012 | 1. single titer ≥ 1:800 OR 2. negative to ≥ 1:200* OR 3. ≥4-fold rising | Co, Ca, Pa, At, Bl, Gr, Cy, Sh, Ta, Br, Cs, Ba, Ce, Dj, He, Ic, Cx, Lo, Pn, Po, Py, Hr, Wo, Cn, Mi | - | - | - | - | [20] |
| Silpasakorn, 2011 | 1. ≥4-fold rising | no data | ✓ | - | - | - | [21] |
| Cohen, 2007 | 1. ≥4-fold rising | no data | - | - | - | - | [22] |
| Blacksell, 2006 | 1. single titer ≥ 1:400 OR 2. ≥4-fold rising | Po, Hr, Ta, Gr, Ce, Co, Au, Py, Ca, He, Mi, Sa, At, Cy, Bl, Ba, Dj, Ja, Pn, Sh, Mw (additional: Br, Ic, Pi, Pr, Sx, Se, Pa, An, Rc, Sm) | - | - | - | - | [23] |
| Sehgal, 2003 | 1. single titer ≥ 1:400 OR 2. negative to ≥ 1:100* OR 3. ≥4-fold rising | Gr, Au, Bl, La, Py, Ta, Po, Ra, Ca, Pi, He, Hr | EMJH | - | - | - | [24] |

* Seroconversion

Serovars abbreviations:

An, Andamana; Au, Australis; At, Autumnalis; Bl, Ballum; Ba, Bataviae; Br, Bratislava; Cn, Canalzonae; Ca, Canicola; Ce, Celledoni; Co, Copenhageni; Cx, Coxi; Cy, Cynopteri; Dj, Djasiman; Gr, ﻿Grippotyphosa; Hr, Hardjo; Ha, Hardjoprajitno; He, Hebdonadis; Ic, Icterohaemorrhagiae; Ja, Javanica; La, Lai; Ls, Losbanos; Lo, Louisaina; Mn, Manhao; Ma, Manilae; Mi, Mini; Pn, Panama; Pa, Patoc; Ph, Pohnpei; Pi, Poi; Po, Pomona; Pr, Proechimys; Py, Pyrogenes; Rc, Rachmati; Ra, Ratnapura; Rn, Ranarum, Sa, Sarmin; Sx, Saxkoebing; Se, Sejroe; Sm, Semaranga; Sh, Shermani; Ta, Tarrasovi; Wo, Wolffi

**References**

1. Jirawannaporn S, Limothai U, Tachaboon S, Dinhuzen J, Kiatamornrak P, Chaisuriyong W, et al. The combination of RPA-CRISPR/Cas12a and Leptospira IgM RDT enhances the early detection of leptospirosis. PLoS Negl Trop Dis. 2023;17: e0011596. doi:10.1371/JOURNAL.PNTD.0011596

2. Campos H de S, da Silva ED, Lima GS de, Resende R de O, Burth P. Leptospira interrogans insoluble fraction as a potential antigen source for lateral flow immunochromatography. Mem Inst Oswaldo Cruz. 2023;118. doi:10.1590/0074-02760220265

3. Bottieau E, Van Duffel L, El Safi S, Koirala KD, Khanal B, Rijal S, et al. Etiological spectrum of persistent fever in the tropics and predictors of ubiquitous infections: a prospective four-country study with pooled analysis. BMC Med. 2022;20: 1–11. doi:10.1186/S12916-022-02347-8

4. Dinhuzen J, Limothai U, Tachaboon S, Krairojananan P, Laosatiankit B, Boonprasong S, et al. A prospective study to evaluate the accuracy of rapid diagnostic tests for diagnosis of human leptospirosis: Result from THAI-LEPTO AKI study. PLoS Negl Trop Dis. 2021;15: e0009159. doi:10.1371/journal.pntd.0009159

5. Silpasakorn S, Kim YW, Suputtamongkol Y. Evaluation of combined rapid immunoglobulin M and immunoglobulin G lateral flow assays for the diagnosis of leptospirosis, scrub typhus, and hantavirus infection. Siriraj Med J. 2020;72: 253–258. doi:10.33192/SMJ.2020.34

6. Dawson P, Marfel M, Galloway R, Tareg A, Paz-Bailey G, Muñoz-Jordán JL, et al. Notes from the field: Interpretation of rapid diagnostic tests for leptospirosis during a dengue outbreak — Yap State, Federated States of Micronesia, 2019. MMWR Morb Mortal Wkly Rep. 2020;69: 1832–1833. doi:10.15585/mmwr.mm6948a6

7. Rao M, Amran F, Aqilla N. Evaluation of a Rapid Kit for Detection of IgM against Leptospira in Human. Can J Infect Dis Med Microbiol. 2019;2019: 5763595. doi:10.1155/2019/5763595

8. Alia SN, Joseph N, Philip N, Azhari NN, Garba B, Masri SN, et al. Diagnostic accuracy of rapid diagnostic tests for the early detection of leptospirosis. J Infect Public Health. 2019;12: 263–269. doi:10.1016/J.JIPH.2018.10.137

9. Amran F, Liow YL, Halim NAN. Evaluation of a commercial immuno-chromatographic assay kit for rapid detection of IgM antibodies against Leptospira antigen in human serum. J Korean Med Sci. 2018;33: e131. doi:10.3346/jkms.2018.33.e131

10. Nabity SA, Hagan JE, Araújo G, Damião AO, Cruz JS, Nery N, et al. Prospective evaluation of accuracy and clinical utility of the Dual Path Platform (DPP) assay for the point-of-care diagnosis of leptospirosis in hospitalized patients. PLoS Negl Trop Dis. 2018;12: e0006285. doi:10.1371/journal.pntd.0006285

11. Doungchawee G, Sutdan D, Niwatayakul K, Inwisai T, Sitthipunya A, Boonsathorn N, et al. Development and evaluation of an immunochromatographic assay to detect serum anti-leptospiral lipopolysaccharide IgM in acute leptospirosis. Sci Rep. 2017;7: 1–9. doi:10.1038/s41598-017-02654-8

12. Lee JW, Park S, Kim SH, Christova I, Jacob P, Vanasco NB, et al. Clinical evaluation of rapid diagnostic test kit using the polysaccharide as a genus-Specific diagnostic antigen for leptospirosis in Korea, Bulgaria, and Argentina. J Korean Med Sci. 2016;31: 183–189. doi:0.3346/jkms.2016.31.2.183

13. Eugene EJ, Handunnetti SM, Wickramasinghe SA, Kalugalage TL, Rodrigo C, Wickremesinghe H, et al. Evaluation of two immunodiagnostic tests for early rapid diagnosis of leptospirosis in Sri Lanka: A preliminary study. BMC Infect Dis. 2015;15: 1–5. doi:10.1186/s12879-015-1080-z

14. Podgoršek D, Cerar T, Logar M, Lešničar G, Remec T, Baklan Z, et al. Evaluation of the immunochromatographic (Leptocheck) test for detection of specific antibodies against leptospires. Wien Klin Wochenschr. 2015;127: 948–953. doi:10.1007/s00508-015-0853-0

15. Niloofa R, Fernando N, de Silva NL, Karunanayake L, Wickramasinghe H, Dikmadugoda N, et al. Diagnosis of Leptospirosis: Comparison between Microscopic Agglutination Test, IgM-ELISA and IgM Rapid Immunochromatography Test. Dellagostin OA, editor. PLoS One. 2015;10: e0129236. doi:10.1371/journal.pone.0129236

16. Colt S, Pavlin BI, Kool JL, Johnson E, McCool JP, Woodward AJ. Human leptospirosis in the federated states of micronesia: A hospital-based febrile illness survey. BMC Infect Dis. 2014;14: 1–6. doi:10.1186/1471-2334-14-186

17. Chang CH, Riazi M, Yunus MH, Osman S, Noordin R. Limited diagnostic value of two commercial rapid tests for acute leptospirosis detection in Malaysia. Diagn Microbiol Infect Dis. 2014;80: 278–281. doi:10.1016/j.diagmicrobio.2014.08.012

18. Widiyanti D, Koizumi N, Fukui T, Muslich LT, Segawa T, Villanueva SYAM, et al. Development of immunochromatography-based methods for detection of leptospiral lipopolysaccharide antigen in urine. Clin Vaccine Immunol. 2013;20: 683–690. doi:10.1128/CVI.00756-12

19. Goris MGA, Leeflang MMG, Loden M, Wagenaar JFP, Klatser PR, Hartskeerl RA, et al. Prospective evaluation of three rapid diagnostic tests for diagnosis of human leptospirosis. PLoS Negl Trop Dis. 2013;7: e2290. doi:10.1371/journal.pntd.0002290

20. Nabity SA, Ribeiro GS, Lessa Aquino C, Takahashi D, Damião AO, Gonçalves AHO, et al. Accuracy of a Dual Path Platform (DPP) assay for the rapid point-of-care diagnosis of human leptospirosis. PLoS Negl Trop Dis. 2012;6: e1878. doi:10.1371/journal.pntd.0001878

21. Silpasakorn S, Waywa D, Hoontrakul S, Suttinont C, Losuwanaluk K, Suputtamongkol Y. Performance of Leptospira immunoglobulin M ELISA and rapid immunoglobulin G immunochromatographic assays for the diagnosis of leptospirosis. J Med Assoc Thai. 2011;94: S203–S206.

22. Cohen AL, Dowell SF, Nisalak A, Mammen MP, Petkanchanapong W, Fisk TL. Rapid diagnostic tests for dengue and leptospirosis: antibody detection is insensitive at presentation. Trop Med Int Health. 2007;12: 47–51. doi:10.1111/j.1365-3156.2006.01752.x

23. Blacksell SD, Smythe L, Phetsouvanh R, Dohnt M, Hartskeerl R, Symonds M, et al. Limited diagnostic capacities of two commercial assays for the detection of Leptospira immunoglobulin M antibodies in Laos. Clin Vaccine Immunol. 2006;13: 1166–1169. doi:10.1128/CVI.00219-06

24. Sehgal SC, Vijayachari P, Sugunan AP, Umapathi T. Field application of Lepto lateral flow for rapid diagnosis of leptospirosis. J Med Microbiol. 2003;52: 897–901.
