# Supplementary material for: Accuracy of rapid lateral flow immunoassays for human leptospirosis diagnosis: A systematic review and meta-analysis
Source: PLoS Negl Trop Dis. 2024 May 15;18(5):e0012174. doi: 10.1371/journal.pntd.0012174 (PMC11132494; doi:10.1371/journal.pntd.0012174)
Supplement: S5 Table — (DOCX) [file pntd.0012174.s007.docx]

**S5 Table** List of LFI evaluated by the studies included in this review

| **Lateral Flow Immunoassay** | **Manufacturer** | **Detection** | **Target** |
| --- | --- | --- | --- |
| Bioline Leptospira IgM | Abbott | IgM | undisclosed |
| TRUSTline | Athenese-Dx, India | IgM/IgG | *L. interrogans* |
| Dual Path Platform | Chembio Diagnostic Systems, USA | antibody | recombinant leptospiral immunoglobulin-like protein |
| Medical Science Public Health LFI | Department of Medical Sciences, Ministry of Public Health, Thailand | IgM | undisclosed |
| ImmuneMed AFI rapid | ImmuneMed AFI rapid | IgM/IgG | *L. patoc* polysaccharide |
| ImmuneMed Leptospira IgM Duo Rapid Test | ImmuneMed AFI rapid | IgM | Possibly *L. patoc* polysaccharide |
| ImmuneMed Leptospira Rapid Test | ImmuneMed AFI rapid | IgM/IgG | Possible *L. patoc* polysaccharide |
| J.Mitra | J.Mitra, India | IgM/IgG | undisclosed |
| Test-IT Leptospira IgM | LifeAssay Diagnostics (Pty) Ltd, South Africa | IgM | undisclosed |
| VISITECT Lepto | Omega Diagnostics Group PLC, UK | IgM | undisclosed |
| LeptoTek Lateral Flow | Organon-Teknika, Netherlands | IgM | undisclosed |
| Multi-Test Dip_S-Ticks, DSLST | PanBio Ltd. Australia and PanBio Inc. USA | IgM | *L. interrogans* and *L. biflexa* |
| SD bioline Leptospira IgG/IgM | Standard Diagnostics, South Korea | IgM/IgG | *L. interrogans* |
| SD bioline leptospirosis | Standard Diagnostics, South Korea | IgG | *L. interrogans* |
| Lepto Lateral flow | The Royal Tropical Institute, The Netherlands | IgM | Patoc 1, heat extract |
| Leptocheck WB | Zephyr Biomedicals, India | IgM | undisclosed |
| LEPkit | in-house (Doungchawee et. al.) | IgM | LPS from six different serovars* |
| IN-LFI | in-house (Campos et. al.) | IgM | Insoluble fraction from lysate of *L. interrogans* serovar *Canicola* |
| dipsticks | in-house (Widiyanti et. al.) | antigen | LPS** |
| ICG-based LFA | in-house (Widiyanti et. al.) | antigen | LPS** |

* Serovars Autumnalis, Bratislava, Canicola, Pomona, Sejroe, and Shermani

** The tests were developed using monoclonal antibody specific to LPS of *L. interrogans* serovar Hebdomad
