# Supplementary material for: Accuracy of rapid lateral flow immunoassays for human leptospirosis diagnosis: A systematic review and meta-analysis
Source: PLoS Negl Trop Dis. 2024 May 15;18(5):e0012174. doi: 10.1371/journal.pntd.0012174 (PMC11132494; doi:10.1371/journal.pntd.0012174)
Supplement: S6 Table — (DOCX) [file pntd.0012174.s008.docx]

**S6 Table** Subgroup analysis by brand of LFI*

| LFIs | Number of data entry | Combined sensitivity (95% confident interval) | Combined specificity (95% confident interval) |
| --- | --- | --- | --- |
| Dual path platform | 5 | 0.90 (0.82 – 0.94) | 0.89 (0.81 – 0.94) |
| ImmueMed AFI rapid | 6 | 0.50 (0.24 – 0.75) | 0.99 (0.97 – 1.00) |
| LeptoTek lateral flow | 4 | 0.65 (0.52 – 0.76) | 0.89 (0.76 – 0.95) |
| Leptocheck WB | 9 | 0.75 (0.66 – 0.82) | 0.88 (0.75 – 0.95) |

*The analysis was limited to LFIs that have four or more data entries
