# Supplementary material for: Accuracy of rapid lateral flow immunoassays for human leptospirosis diagnosis: A systematic review and meta-analysis
Source: PLoS Negl Trop Dis. 2024 May 15;18(5):e0012174. doi: 10.1371/journal.pntd.0012174 (PMC11132494; doi:10.1371/journal.pntd.0012174)
Supplement: S7 Table — (DOCX) [file pntd.0012174.s009.docx]

**S7 Table** Subgroup analysis by detection targets

| **Detection** | **Number of data entry** | **Combined sensitivity (95% CI)** | **Combined specificity (95% CI)** |
| --- | --- | --- | --- |
| IgM | 29 | 0.69 (0.58 – 0.78) | 0.90 (0.86 – 0.93) |
| IgG | 4 | 0.23 (0.03 – 0.73) | 1.00 (0.94 – 1.00) |
| IgM/IgG | 9 | 0.62 (0.26 – 0.88) | 0.97 (0.94 – 0.98) |

Entry ID 3-7 and 46-47 were not included in the analysis because they are antigen detection LFIs, or type of antibody detected is not revealed.
