# Supplementary material for: Accuracy of rapid lateral flow immunoassays for human leptospirosis diagnosis: A systematic review and meta-analysis
Source: PLoS Negl Trop Dis. 2024 May 15;18(5):e0012174. doi: 10.1371/journal.pntd.0012174 (PMC11132494; doi:10.1371/journal.pntd.0012174)
Supplement: S8 Table — (DOCX) [file pntd.0012174.s010.docx]

**S8 Table** Subgroup analysis by phases of infection

| **Phase of infection** | **Number of data entry** | **Combined sensitivity (95% CI)** | **Combined specificity (95% CI)** |
| --- | --- | --- | --- |
| Acute | 29 | 0.67 (0.50 – 0.80) | 0.93 (0.88 – 0.96) |
| Convalescent | 9 | 0.64 (0.39 – 0.83) | 0.94 (0.89 – 0.97) |
| Mixed | 6 | 0.69 (0.56 – 0.80) | 0.94 (0.85 – 0.97) |

Entry ID 46, 47 were not included in the analysis because they are antigen detection LFIs.

Entry ID 1, 18, 38 were not included because phase of infection is not specified.
