# Supplementary figures and images for: Accuracy of rapid lateral flow immunoassays for human leptospirosis diagnosis: A systematic review and meta-analysis
Source: PLoS Negl Trop Dis. 2024 May 15;18(5):e0012174. doi: 10.1371/journal.pntd.0012174 (PMC11132494; doi:10.1371/journal.pntd.0012174)

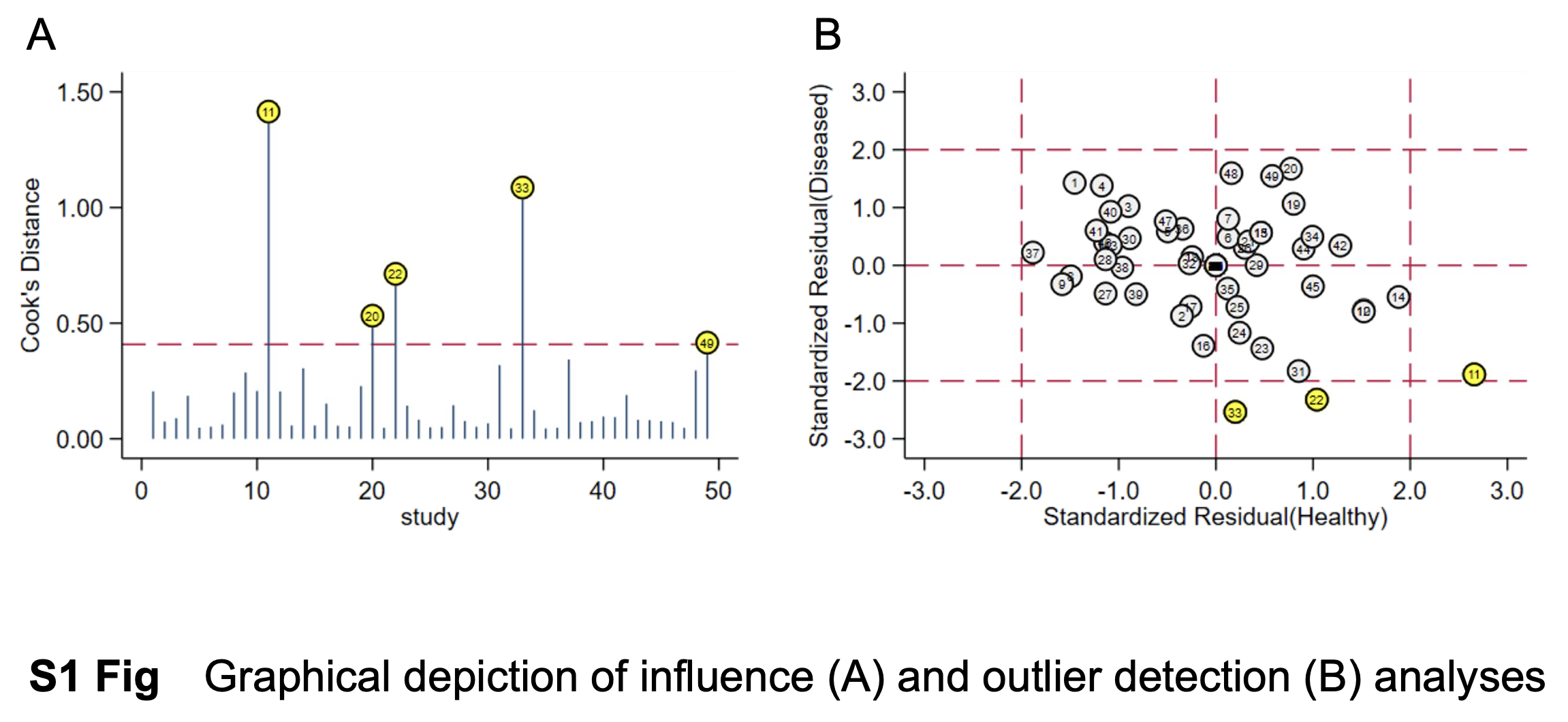

Supplement: S1 Fig — Graphical depiction of influence (A) and outlier detection (B) analyses. (TIFF) [file pntd.0012174.s011.tiff]

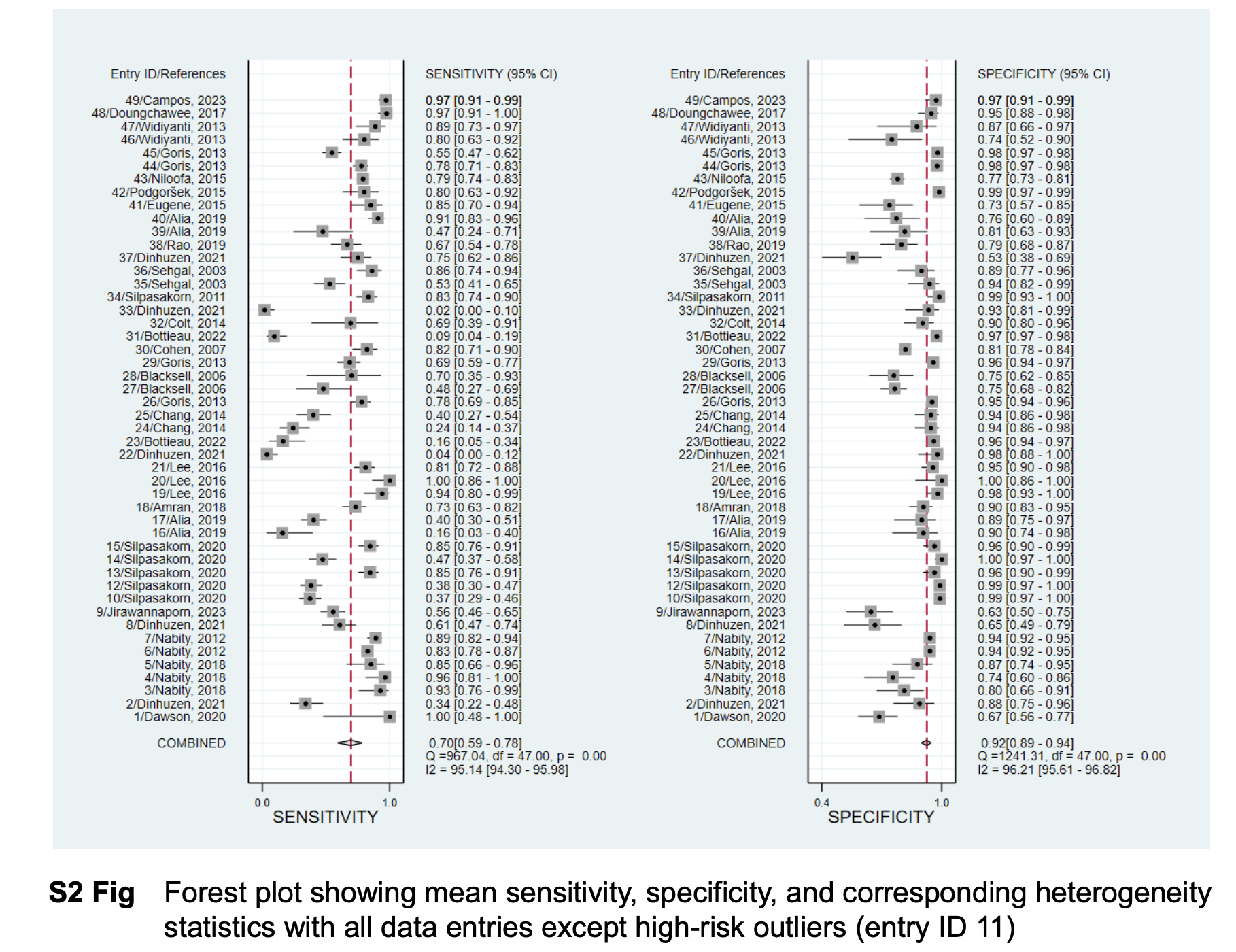

Supplement: S2 Fig — (TIFF) [file pntd.0012174.s012.tiff]

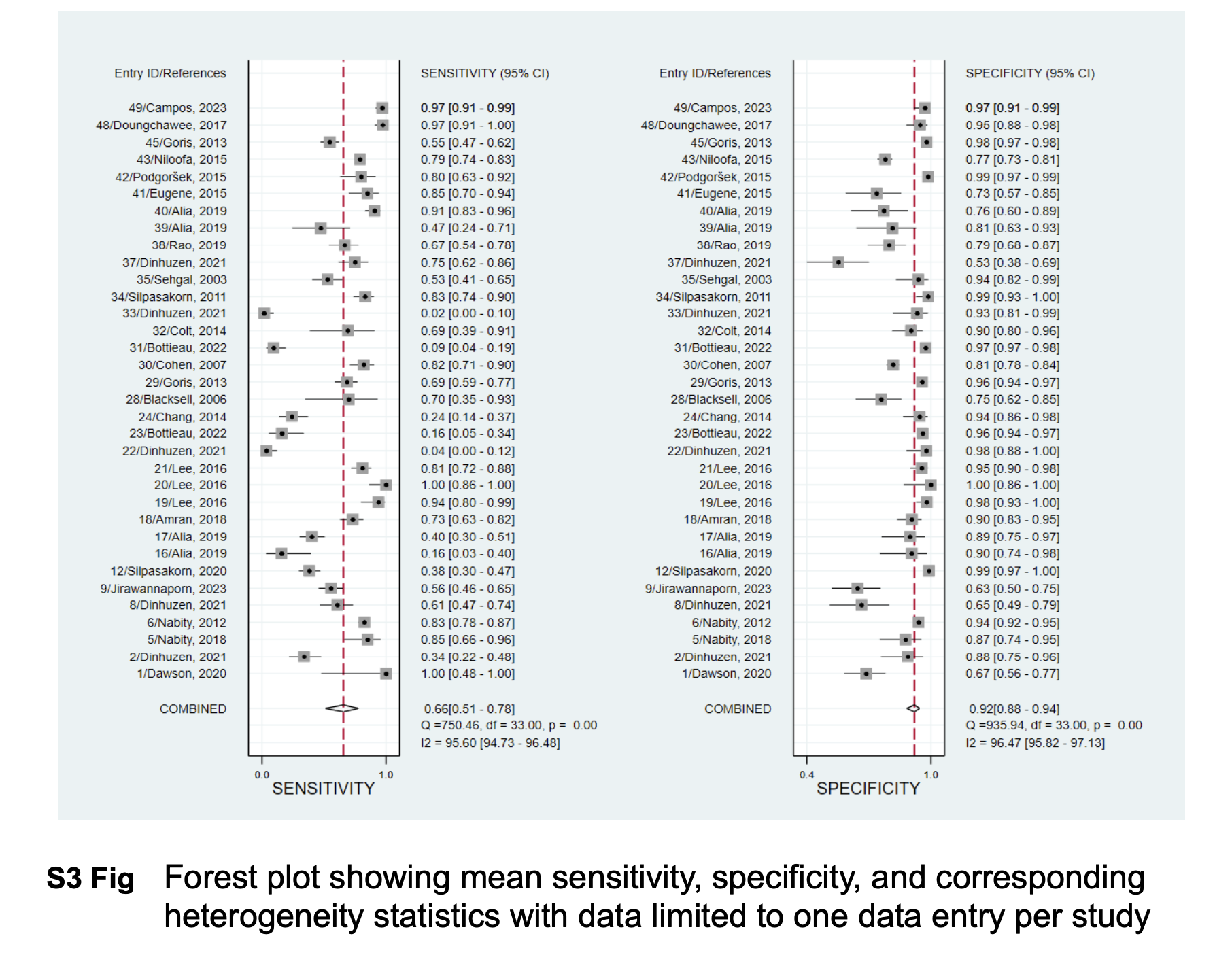

Supplement: S3 Fig — (TIFF) [file pntd.0012174.s013.tiff]
