# Supplementary material for: Accuracy of rapid lateral flow immunoassays for human leptospirosis diagnosis: A systematic review and meta-analysis
Source: PLoS Negl Trop Dis. 2024 May 15;18(5):e0012174. doi: 10.1371/journal.pntd.0012174 (PMC11132494; doi:10.1371/journal.pntd.0012174)
Supplement: S1 Appendix — (DOCX) [file pntd.0012174.s014.docx]

**S1 Appendix** Search strategy

Key concepts and search strategy of three standard databases

| **Key concepts** | **Keywords** | **MeSH terms** | **EMTREE terms** |
| --- | --- | --- | --- |
| #1 Domain | Leptospirosis, Leptospira, Leptospiral, Leptospires | Leptospirosis, Leptospira | Leptospirosis, Leptospira |
| #2 Diagnostic test | Lateral flow assay, Rapid test, Dip strip, Dipstick, Immunochromatographic assay, Rapid diagnosis | Diagnostic test, Immunoassay | Lateral flow immunochromatography, Immunochromatographic assay kit, Diagnostic test, Rapid test |
| #3 Outcome | Sensitivity and specificity, Diagnostic accuracy, Validity | Sensitivity and specificity, ROC curve, Predictive Value of tests | Diagnostic accuracy  Sensitivity and specificity |

Summary of strategy: Last updated date on Jan 28, 2024

| **Key concepts** | **Search terms** | **N = 459** |
| --- | --- | --- |
| PubMed search | ("leptospir*"[Title/Abstract] OR "leptospir*"[MeSH Terms]) AND ("lateral flow assay"[Text Word] OR "rapid test"[Text Word] OR "dip strip"[Text Word] OR "Dipstick"[Text Word] OR "immunochromatographic assay"[Text Word] OR "rapid diagnosis"[Text Word] OR "diagnostic tests, routine"[MeSH Terms] OR "immunoassay"[MeSH Terms]) AND (("Sensitivity"[Text Word] AND "Specificity"[Text Word]) OR "diagnostic accuracy"[Text Word] OR "Validity"[Text Word] OR (("hypersensitivity"[MeSH Terms] OR "hypersensitivity"[All Fields] OR "sensitive"[All Fields] OR "sensitively"[All Fields] OR "sensitives"[All Fields] OR "sensitivities"[All Fields] OR "sensitivity and specificity"[MeSH Terms] OR ("Sensitivity"[All Fields] AND "Specificity"[All Fields]) OR "sensitivity and specificity"[All Fields] OR "Sensitivity"[All Fields]) AND "sensitivity and specificity"[MeSH Terms]) OR "roc curve"[MeSH Terms] OR "predictive value of tests"[MeSH Terms]) | 275 |
| EMBASE search on Ovid | (leptospir*.ab,kf,kw,ti.) AND ( (lateral flow assay.ab,kf,kw,ti.) OR (rapid test.ab,kf,kw,ti.) OR (dip strip.ab,kf,kw,ti.) OR (immunochromatographic assay.ab,kf,kw,ti.) OR (rapid diagnosis.ab,kf,kw,ti.) OR (diagnostic test.ab,kf,kw,ti.) OR immunochromatographic assay kit.ab,kf,kw,ti.) ) AND ( ((sensitivity and specificity).ab,kf,kw,ti.)) OR (diagnostic accuracy.ab,kf,kw,ti.) OR (validity.ab,kf,kw,ti.) ) | 93 |
| Scopus search | ( ( TITLE-ABS-KEY ( "Validity" ) ) OR ( TITLE-ABS-KEY ( "Diagnostic accuracy" ) ) OR ( TITLE-ABS-KEY ( "sensitivity and specificity" ) ) ) AND ( ( TITLE-ABS-KEY ( "Rapid diagnosis" ) ) OR ( TITLE-ABS-KEY ( "Immunochromatographic assay" ) ) OR ( TITLE-ABS-KEY ( "Dip strip" ) ) OR ( TITLE-ABS-KEY ( "Rapid test" ) ) OR ( TITLE-ABS-KEY ( "Lateral flow assay" ) ) ) AND ( TITLE-ABS-KEY ( "leptospir*" ) ) | 91 |
