# Supplementary material for: Accuracy of rapid lateral flow immunoassays for human leptospirosis diagnosis: A systematic review and meta-analysis
Source: PLoS Negl Trop Dis. 2024 May 15;18(5):e0012174. doi: 10.1371/journal.pntd.0012174 (PMC11132494; doi:10.1371/journal.pntd.0012174)
Supplement: S2 Appendix — (DOCX) [file pntd.0012174.s015.docx]

**﻿Jirawannaporn, 2023**

**﻿The combination of RPA-CRISPR/Cas12a and Leptospira IgM RDT enhances the early detection of leptospirosis**

Patient recruitment

This study was conducted in Thailand using samples from two cohorts. The first group of samples (110 samples) were ﻿taken from previous research that were performed between December 2015 and November 2016. The second group of samples (61 samples) were collected between January 2019 to January 2022. Patients with the age of 18 or older who were admitted to hospitals with clinical suspicion of leptospirosis (body temperature higher than 38 °C and history of exposure to reservoir animals or flood water) were enrolled to the study. However, patients who suffered from other known infectious diseases were excluded. Blood samples were collected on the first day of enrollment and day 7 after enrollment ﻿for pair serum MAT testing.

Reference tests

In this study, MAT, direct culture, and real-time PCR were used to define leptospirosis status of all 171 enrolled patients using ﻿samples from the first day of enrollment. Patients were defined as confirmed leptospirosis if:

1. **MAT** assay result showed:
2. a single serum titer of ≥ 1:800 OR
3. a ≥ four-fold rise in pair serum (seven days apart) OR
4. **Real-time PCR** targeting LipL32 gene yielded a positive result OR
5. **Direct blood culture** using EMJH medium (30 °C) showed the growth of leptospires

MAT conducted in this study used ﻿ 24 serovars of *Leptospira interrogans* including Australis, Autumnalis, Ballum, Bataviae, Canicola, Cellidoni, Cynopteri, Djasiman, Grippotyphosa, Hebdonadis, Icterohaemorrhagiae, Javanica, Louisiana, Manhao, Mini, Panama, Pomona, Pyrogenes, Ranarum, Sarmin, Sejroe, Shermani, Tarasovi, and Semaranga.

Of 171 patients, the results showed that 106 patients were confirmed leptospirosis.

Among 106 sera that were confirmed leptospirosis, MAT positive = 39, culture positive = 4, PCR positive = 88.

Index tests

The index test evaluated in this study was **Medical Science Public Health** (Department of Medical Sciences, Ministry of Public Health, Thailand). The test was designed to detect IgM specific to *Leptospira*. The evaluation was performed as described in the manufacturer’s manual with both acute-phase samples and convalescent samples and in a blinded manner.

Results

|  | **Medical Science Public Health** |
| --- | --- |
| True positive | 59 |
| False positive | 24 |
| True negative | 41 |
| False negative | 47 |
| total | 171 |
| **Sensitivity** | **55.67** |
| **Specificity** | **63.08** |

**Campos, 2023**

**﻿Leptospira interrogans insoluble fraction as a potential antigen source for lateral flow immunochromatography**

Patient recruitment

This study was conducted in Brazil. Upon recruitment, samples were placed into groups based on their immune profile as the following:

Group 1: N = 100; Patients with leptospirosis in acute phase with positive diagnosis, determined by MAT

Group 2: N = 100; Patients with acute febrile illness from endemic area for leptospirosis with negative diagnosis, determined by MAT

Group 3: N = 60; ﻿Patients hospitalised with leptospirosis in convalescent phase, with positive diagnosis, determined by MAT

Group 4: N = 45; ﻿Patients with hepatitis C (HCV) (10), hepatitis B (HBV) (10), HIV (10) and syphilis (15) from a sera panel

Reference tests

In this study, MAT were used to define leptospirosis status of patients but the definition and serovars used was not reported.

Index tests

The index test evaluated in this study was an in-house **IN-LFI**, which was developed using insoluble fraction (IN) derived from ﻿*L. interrogans* ﻿serovar *Canicola* extract. The test was designed to detect IgM specific to *Leptospira*.

Results: only the result derived from acute samples (group 1 and 2) was calculated

|  | **IN-LFI** |
| --- | --- |
| True positive | 97 |
| False positive | 3 |
| True negative | 97 |
| False negative | 3 |
| total | 200 |
| **Sensitivity** | **97.0** |
| **Specificity** | **97.0** |

**Bottieau, 2022**

**﻿Etiological spectrum of persistent fever in the tropics and predictors of ubiquitous infections: a prospective four-country study with pooled analysis**

Patient recruitment

This is a multicenter prospective study conducted in four countries including Sudan, Congo, Nepal and Cambodia between January 2013 to October 2014. Patients with the age of five or older (or 18 or older for Cambodia) who presented with persistent fever (seven days or more) were enrolled to the study. However, patients who i) already had a laboratory-confirmed diagnosis at the time of consultation, ii) were already admitted for more than 48 hours for another reason or iii) presented with conditions that require immediate intensive care were excluded from the study. After enrollment and clinical examination, blood and urine samples were collected. Follow-up took place within 1 month after enrollment to allow paired serology test. Of 1,939 enrolled patients, 1,922 had sufficient data for analysis.

Reference tests

In this study, MAT and PCR were used to define leptospirosis status of all 1,922 patients. Patients were defined as confirmed leptospirosis if:

1. **PCR** assay in blood or urine samples yielded a positive result. OR
2. **MAT** assay result showed a ≥ four-fold rise in paired serum. *Note:* *the diagnosis was considered as probable in case of antibody test positive on single serum*.

Serovar of *Leptospira* spp. included in MAT was not reported.

Of 1,922 patients, the results showed that 31 patients were confirmed leptospirosis and 46 patients were considered as probable.

Index tests

Two commercial lateral flow immunoassays (LFI) were evaluated:

1. **Test-IT Leptospira IgM** (LifeAssay Diagnostics (Pty) Ltd, South Africa)

This assay was designed to detect IgM specific to *Leptospira*.

1. **SD bioline Leptospira IgG/IgM** (Standard Diagnostics, South Korea)

This assay was designed to detect IgM and IgG specific to *L. interrogans*. The assay was considered positive if any line (IgM or IgG) was positive.

The evaluations were performed using serum samples collected from the first day of enrollment. The duration of fever of enrolled patients (mean, [IQR]) was 14 days, [9-28 days].

The technicians performing the LFIs were blinded to any reference test results.

Results

|  | **SD bioline Leptospira IgG/IgM** | **Test-IT Leptospira IgM** |
| --- | --- | --- |
| True positive | 6 | 5 |
| False positive | 58 | 26 |
| True negative | 1,804 | 785 |
| False negative | 49 | 34 |
| total | 1,917 | 850 |
| **Sensitivity** | **9 (4-19)** | **16 (7-33)** |
| **Specificity** | **97.4 (96.5 – 98.4)** | **95.8 (94.3-98.2)** |

**Dinhuzen, 2021**

**﻿A prospective study to evaluate the accuracy of rapid diagnostic tests for diagnosis of human leptospirosis: Result from THAI-LEPTO AKI study**

Patient recruitment

This is a prospective study conducted in Thailand between December 2015 to November 2016. Patients with the age of 18 or older who were leptospirosis-suspected (had body temperature higher than 38 °C and history of exposure to reservoir animals or flood water) were enrolled to the study. However, patients who suffered from other known infectious diseases were excluded. Blood samples were collected on the first day of enrollment and day 7 after enrollment. Of 330 enrolled patients, only 99 stored sera were randomly selected for the study.

Reference tests

In this study, MAT, direct culture and real-time PCR were used to define leptospirosis status of all 330 enrolled patients. Patients were defined as confirmed leptospirosis if:

1. **MAT** assay result showed:
2. a single serum titer of ≥ 1:400 OR
3. a ≥ four-fold rise in pair serum (seven days apart) OR
4. **Real-time PCR** targeting LipL32 gene yielded a positive result OR
5. **Direct blood culture** using EMJH medium (30 °C for 2 weeks) showed the growth of leptospires

MAT conducted in this study used at least 7 serovars of *Leptospira interrogans* including Shermani, Australis, Sejroe, Louisaina, ﻿Grippotyphosa, Autumnalis and Semaranga.

Of 330 patients, the results showed that 228 patients were confirmed leptospirosis.

Among 99 selected sera, 56 were confirmed leptospirosis (MAT positive = 29, culture positive = 5, PCR positive = 52).

Index tests

Five in-house and commercial lateral flow immunoassays (LFI) were evaluated:

1. **Medical Science Public Health** (Department of Medical Sciences, Ministry of Public Health, Thailand) *defined as RTD1*

This assay was designed to detect IgM specific to *Leptospira*.

1. **Leptocheck WB** (Zephyr Biomedicals, India) *defined as RTD2*

This assay was designed to detect IgM specific to *Leptospira*.

1. **SD bioline leptospirosis** (Standard Diagnostics, South Korea) *defined as RTD3*

This assay was designed to detect IgG specific to *L. interrogans*.

1. **TRUSTline** (Athenese-Dx, India) *defined as RTD4*

This assay was designed to simultaneously and differentially detect IgM and IgG specific to *L. interrogans*.

1. **J.Mitra** (J.Mitra, India) *defined as RTD5*

This assay was designed to simultaneously and differentially detect IgM and IgG specific to *Leptospira*.

The evaluations were performed using serum samples collected at the first day of enrollment which include both acute (within 7 days of fever onset) and convalescence (> 7 days of fever onset) sera. Each test interpreted by 3 certified technicians. The tests were considered positive if at least 2 of 3 technicians called the results as positive.

Results

|  | **RTD1** | **RTD2** | **RTD3** | **RTD4** | **RTD5** |
| --- | --- | --- | --- | --- | --- |
| True positive | 34 | 42 | 1 | 19 | 2 |
| False positive | 22 | 14 | 55 | 37 | 54 |
| True negative | 28 | 23 | 40 | 38 | 42 |
| False negative | 15 | 20 | 3 | 5 | 1 |
| total | 99 | 99 | 99 | 99 | 99 |
| **Sensitivity** | **60.70** | **75.00** | **1.80** | **33.90** | **3.60** |
| **Specificity** | **65.10** | **53.50** | **93.00** | **88.40** | **97.70** |

**Silpasakorn, 2020**

**﻿** **﻿Evaluation of combined rapid immunoglobulin M and immunoglobulin G lateral flow assays for the diagnosis of leptospirosis, scrub typhus, and hantavirus infection**

Sample selection

This is a retrospective study aimed to evaluate the accuracy of lateral flow immunoassays using archived serum samples collected in Thailand. There were 2 groups of samples selected for the study.

1. **Leptospirosis-suspected sample group**

This group contained 259 sera collected from adult patients (>18 years) who presented with acute fever (oral temperature > 38 °C for less than 15 days) between January 2000 and December 2018. Blood samples were collected on the day of admission and/or during convalescence or after discharge from the hospital.

1. **Non-leptospirosis sample group**

This group contained 175 sera collected from patients with other febrile illnesses including dengue infection (n = 59), zika virus infection (n = 16), influenza A or influenza B (n = 26), murine typhus (n = 60), bacterial infections such as *Escherichia coli* septicemia, melioidosis and salmonellosis (n = 8) and *Plasmodium falciparum* (n = 6). All these samples were tested by IFA and shown to be negative for *Leptospira* and *O. tsutsugamushi*.

Reference tests

In this study, **IFA** was used to define leptospirosis status of all 434 patients. Patients were defined as confirmed leptospirosis if IFA results showed:

1. a single serum titer of ≥ 1:400 OR
2. a ≥ four-fold rise in paired serum

IFA was performed using *Leptospira interrogans* serovar autumnalis as the antigen.

Among all these 434 patients, the IFA results showed that 131 patients were confirmed leptospirosis. Of these 131 patients, convalescent samples were available in 91 patients.

Index tests

The index test evaluated in this study was **ImmuneMed AFI rapid** (ImmuneMed, South Korea). The test was designed for differential IgM and IgG detection against polysaccharide antigen of nonpathogenic *Leptospira patoc*. The evaluation was performed as described in the manufacturer’s manual with both acute-phase samples and convalescent samples.

Results

|  | **ImmuneMed AFI rapid** | | | | | |
| --- | --- | --- | --- | --- | --- | --- |
|  | **Acute-phase samples** | | | **Convalescent phase samples** | | |
|  | **IgM** | **IgG** | **IgM/IgG** | **IgM** | **IgG** | **IgM/IgG** |
| True positive | 49 | 12 | 50 | 77 | 43 | 77 |
| False positive | 82 | 119 | 81 | 14 | 48 | 14 |
| True negative | 300 | 303 | 300 | 100 | 104 | 100 |
| False negative | 3 | 0 | 3 | 4 | 0 | 4 |
| total | 434 | 434 | 434 | 195 | 195 | 195 |
| **Sensitivity** | **37.4**  **(29.1-45.7)** | **9.2**  **(4.3-14.1)** | **38.2**  **(29.9-46.5)** | **84.6**  **(77.1-92)** | **47.3**  **(37-57.5)** | **84.6**  **(77.1-92)** |
| **Specificity** | **99.0**  **(97.9-100)** | **100.0**  **(88.2-100)** | **99.0**  **(97.9-100)** | **96.2**  **(92.5-99.8)** | **100.0** | **96.2**  **(92.5-99.8)** |

**Dawson, 2020**

**﻿** **﻿Interpretation of rapid diagnostic tests for leptospirosis during a dengue outbreak —Yap State, Federated States of Micronesia, 2019**

Patient recruitment

This study was conducted in the Federated States of Micronesia during the concurrent outbreak of dengue and leptospirosis, two infectious diseases that are symptomatically comparable. During May to September 2019, paired acute-phase and convalescent phase sera were collected from patients with dengue-like illness (DLI). DLI was defined as fever and two or more of the following: nausea/vomiting, rash, aches and pain, and any severe dengue warning signs. Among patients recruited during this period, 91 patients were tested for leptospirosis with both LFI and MAT.

Reference tests

In this study, **MAT**, were used to define leptospirosis status of 91 patients. Patients were defined as confirmed leptospirosis if:

1. a single serum titer of ≥ 1:800 OR
2. a ≥ four-fold rise in pair serum

A panel of serovar of *Leptospira* spp. used in MAT was not reported.

Of 91 patients, the results showed that 5 patients were confirmed leptospirosis.

Index tests

The index test evaluated in this study was **Bioline Leptospira IgM** (Abbott). The test was designed for detection of IgM against *Leptospira*.

Results

|  | **Bioline Leptospira IgM** |
| --- | --- |
| True positive | 5 |
| False positive | 28 |
| True negative | 58 |
| False negative | 0 |
| total | 91 |
| **Sensitivity** | **100**  **(57-100)** |
| **Specificity** | **67**  **(57-76)** |

**Rao, 2019**

**﻿Evaluation of a Rapid Kit for Detection of IgM against Leptospira in Human**

Sample selection

This is a retrospective study aimed to evaluate the accuracy of lateral flow immunoassays using archived serum samples collected from various hospitals localized in Malaysia. They were sera from leptospirosis-suspected patients sent to Institute for Medical Research (IMR) for laboratory diagnosis of leptospirosis. A total of 142 sera were used in this evaluation.

Reference tests

In this study, **MAT** was used to define leptospirosis status of all 142 achieved sera samples. Sera were considered positive for leptospirosis if MAT results showed:

1. a single serum titer of ≥ 1:400 OR
2. a ≥ four-fold rise in paired serum

MAT was performed using 20 serovars recommended by WHO and 6 local isolates. The reference strains obtained from the WHO Collaborative Centre for Leptospirosis include Australis, Autumnalis, Bataviae, Ballum, Canicola, Grippotyphosa, Icterohaemorrhagiae, Javanica, Pomona, Pyrogenes, Tarrasovi, Sejroe, and Patoc.

Among all these 142 patients, the MAT results showed that 66 sera samples were confirmed leptospirosis.

Index tests

The index test evaluated in this study was **Leptocheck WB** (Zephyr Biomedical, India). The test was designed for detection of IgM against *Leptospira.* The evaluation was performed as described in the manufacturer’s manual.

Results

|  | **Bioline Leptospira IgM** |
| --- | --- |
| True positive | 44 |
| False positive | 16 |
| True negative | 60 |
| False negative | 22 |
| total | 142 |
| **Sensitivity** | **66.6** |
| **Specificity** | **78.9** |

**Alia, 2019**

**﻿** **Diagnostic accuracy of rapid diagnostic tests for the early detection of leptospirosis**

This study aimed to evaluate the accuracy of lateral flow immunoassays using 2 sets of samples.

**THE FIRST SET OF SAMPLES**

Patient recruitment

The first set of samples used in this study was prospectively collected. The study was conducted at the Serdang Hospital, Malaysia from June 2016 to June 2017. Patients clinically suspected of leptospirosis (acute febrile illness) were enrolled for the study while the patients that showed clear symptoms or confirmed for other illness were excluded. Blood samples were collected on the day of admission. For some patients, samples were also collected on the discharge day (4 days after admission). Among 50 patients recruited, 19 patients were confirmed leptospirosis.

Reference tests

For this set of samples, **MAT** and **qPCR** were used to define leptospirosis status of all 50 patients recruited. Patients were considered positive for leptospirosis if:

1. **MAT** assay result showed:
2. a single serum titer of ≥ 1:400 OR
3. a ≥ four-fold rise in pair serum (seven days apart) OR
4. **Real-time PCR** targeting LipL32 gene yielded a positive result

MAT was performed using 20 serovars of Leptospires. The reference serovars obtained from the WHO Collaborative Centre for Leptospirosis include Australis, Autumnalis, Bataviae, Canicola, Celledoni, Grippotyphosa, Hardjoprajitno, Icterohaemorrhagiae, Javanica, Pyrogenes, Tarrasovi, Djasiman, Pomona and Patoc. In addition to 14 international serovars, 6 local serovars were included.

Among all these 50 patients, 6 were confirmed leptospirosis by MAT and 13 were confirmed leptospirosis by qPCR, giving a total of 13 leptospirosis-confirmed cases.

**THE SECOND SET OF SAMPLES**

Sample selection

The second set of samples used in this study are retrospective samples obtained from Public Health Laboratory of the Kelantan State Health Department, Malaysia. All the samples obtained (n = 135) were from patients with acute leptospirosis (confirmed by MAT, n = 97), other illness and healthy (n = 38).

Reference tests

For this set of samples, **MAT** was used to define leptospirosis status of all 135 samples. Patients were considered positive for leptospirosis if MAT assay results showed a single serum titer of ≥ 1:400. MAT was performed using the same serovars described above.

Index tests

Two commercial lateral flow immunoassays (LFI) were evaluated:

1. **Leptocheck WB** (Zephyr Biomedicals, India)

This assay was designed to detect IgM specific to *Leptospira.*

1. **ImmuneMed Leptospira IgM Duo Rapid Test** (ImmuneMed, South Korea)

This assay was designed to detect IgM specific to *Leptospira* (possibly polysaccharide antigen of nonpathogenic *Leptospira patoc*) at two titers (1:50 and 1:200).

The tests were performed as described in the manufacturer’s manual.

Results

|  | **Prospective study** | | **Retrospective study** | |
| --- | --- | --- | --- | --- |
|  | **Leptocheck WB** | **ImmuneMed Leptospira IgM Duo Rapid Test** | **Leptocheck WB** | **ImmuneMed Leptospira IgM Duo Rapid Test** |
| True positive | 9 | 3 | 88 | 39 |
| False positive | 6 | 3 | 9 | 9 |
| True negative | 25 | 38 | 29 | 29 |
| False negative | 10 | 16 | 9 | 58 |
| total | 50 | 50 | 135 | 135 |
| **Sensitivity** | **47.37** | **15.79** | **90.72** | **40.21** |
| **Specificity** | **80.65** | **90.32** | **76.32** | **89.47** |

**Amran, 2019**

**﻿** **Evaluation of a Commercial ImmunoChromatographic Assay Kit for Rapid Detection of IgM Antibodies against Leptospira Antigen in Human Serum**

Sample selection

This is a retrospective study aimed to evaluate the accuracy of lateral flow immunoassays using archived serum samples collected from various hospitals localized in Malaysia. All these sera were from inpatients with acute febrile illness and leptospirosis-suspected symptoms. From these, a total of 197 serum samples were randomly selected for the study.

Reference tests

In this study, **MAT** was used to define leptospirosis status of the 197 selected sera samples. Sera were considered positive for leptospirosis if MAT results showed:

1. a single serum titer of ≥ 1:400 OR
2. a ≥ four-fold rise in paired serum

MAT was performed using 20 serovars recommended by WHO and 6 local isolates. The reference strains obtained from the WHO Collaborative Centre for Leptospirosis include Australis, Autumnalis, Bataviae, Ballum, Canicola, Grippotyphosa, Icterohaemorrhagiae, Javanica, Pomona, Pyrogenes, Tarrasovi, Sejroe, and Patoc.

Among all these 197 samples, the MAT results showed that 93 sera samples were confirmed leptospirosis.

Index tests

The index test evaluated in this study was **ImmuneMed Leptospira IgM Duo Rapid Test** (ImmuneMed, South Korea). The test was designed for detection of IgM against *Leptospira* (possibly polysaccharide antigen of nonpathogenic *Leptospira patoc*) at 2 at two titers (1:50 and 1:200). The evaluation was performed as described in the manufacturer’s manual. Intermediate results (positive at 1:50 titer only) were considered negative for the test evaluation.

Results

|  | **ImmuneMed Leptospira IgM Duo Rapid Test** |
| --- | --- |
| True positive | 68 |
| False positive | 10 |
| True negative | 94 |
| False negative | 25 |
| total | 197 |
| **Sensitivity** | **73** |
| **Specificity** | **90** |

**Nabity, 2018**

**﻿** **﻿** **Prospective evaluation of accuracy and clinical utility of the Dual Path Platform (DPP) assay for the point-of-care diagnosis of leptospirosis in hospitalized patients**

Patient recruitment

This is a prospective study conducted in Brazil from April to October 2012. Patients were enrolled to the study if they had an acute fever (≥38°C) and presented with ≥1 of the following: acute renal failure; jaundice; acute hepatitis; spontaneous hemorrhage; enteric fever; bilateral conjunctival suffusion; aseptic meningitis; or undifferentiated fever. Patients with aseptic meningitis were also enrolled to the study; however, this is outside the scope of this review. Patients who were younger than 5 years and those were unavailable for clinical evaluation (death or discharge) were excluded. From a total of 108 patients that met all the inclusion criteria, 98 were enrolled to the study (classic leptospirosis group, n = 76 and meningitis group, n = 22). At the enrollment, 3 types of specimens including finger stick blood (FBS), venous whole blood (VWB) and serum were collected. Based upon patient availability, convalescent sera were also collected, generally 15-30 days after admission. Only 76 of these were classified to.

Reference tests

In this study, MAT and blood culture were used to define leptospirosis status of 98 patients. Patients were defined as confirmed leptospirosis if:

1. **Blood culture** using EMJH medium showed the growth of leptospires OR
2. **MAT** assay result showed:
3. a single serum titer of ≥ 1:800 (an MAT titer of 1:200 or 1:400 was defined as probable leptospirosis) OR
4. a seroconversion (undetectable acute-phase tier and convalescent phase titer ≥ 1:200)
5. a ≥ four-fold rise in pair serum (seven days apart) OR

MAT panel included 10 *Leptospira* representing 8 serovars and 8 serogroups. List of serovar used in MAT was not reported.

Of 76 patients enrolled as classic leptospirosis, 27 were confirmed leptospirosis and 2 were probable leptospirosis (which were also excluded from the study).

Index tests

The index test evaluated in this study was **Dual Path Platform** (DPP, Chembio Diagnostic Systems, USA). The test was designed for detection of antibody against recombinant leptospiral immunoglobulin-like (rLig) antigen. To evaluate the accuracy of the test, DPP assays were performed per manufacturer instruction using 3 different types of specimens (FSB, VWB and serum) collected from the enrolled patients. Interpretation of the assays was performed using 3 independent interpreters; the result was determined by concordance of ≥2 interpreters. All interpreters were blinded to all confirmatory diagnostics and clinical data.

Results

|  | **Dual Path Platform** | | |
| --- | --- | --- | --- |
|  | **FSB** | **VWB** | **Serum** |
| True positive | 25 | 26 | 23 |
| False positive | 9 | 12 | 6 |
| True negative | 37 | 35 | 41 |
| False negative | 2 | 1 | 4 |
| total | 73 | 74 | 74 |
| **Sensitivity** | **92.6**  **(75.7-99.1)** | **96.3**  **(81.0-99.4)** | **85.2**  **(66.3-95.7)** |
| **Specificity** | **80.4**  **(66.1-90.6)** | **75.4**  **(59.6-86.0)** | **87.2**  **(74.2-95.1)** |

**Doungchawee, 2017**

**﻿** **﻿** **Development and evaluation of an immunochromatographic assay to detect serum anti-leptospiral lipopolysaccharide IgM in acute leptospirosis**

Sample selection

This is a retrospective study aimed to evaluate the accuracy of lateral flow immunoassays using archived reference serum samples collected in Thailand. There were 2 groups of samples selected for the study.

1. **Leptospirosis reference sample group**

This group contained 77 samples of acute leptospirosis sera (collected within 7 days after disease onset). Gold standard test used for the diagnosis of leptospirosis were culture and MAT.

1. **Non-leptospirosis sample group**

This negative control group consisted of 91 sera samples. Of these, 39 were from healthy blood donors. The remaining 52 samples were from patients with non-leptospirosis including syphilis (n = 12), hepatitis (n = 10), dengue virus infection (n = 10), Scrub typhus (n = 10) and melioidosis (n = 10).

Reference tests

In this study, **Culture** and **MAT** were used to define leptospirosis status of the leptospirosis reference samples. Samples were defined as confirmed leptospirosis if:

1. **Culture** showed the growth of leptospires OR
2. **MAT** assay result showed:
3. a single serum titer of ≥ 1:400 OR
4. a seroconversion (undetectable acute-phase tier and convalescent phase titer ≥ 1:100)
5. a ≥ four-fold rise in pair serum

MAT panel included 26 leptospiral serogroups. List of serovar used in MAT was not reported.

Index tests

The index test evaluated in this study was **LEPkit** which was developed in-house by the investigators. The test was designed for detection of IgM against lipopolysaccharide (LPS) extracted from six different *Leptospira* serovars of local prevalence (Autumnalis, Bratislava, Canicola, Pomona, Sejroe and Shermani).

Results

|  | **LEPkit** |
| --- | --- |
| True positive | 75 |
| False positive | 5 |
| True negative | 86 |
| False negative | 2 |
| total | 168 |
| **Sensitivity** | **97.4 (90.1 - 99.5)** |
| **Specificity** | **94.5 (87.1-98.0)** |

**Lee, 2016**

**﻿** **﻿** **Clinical Evaluation of Rapid Diagnostic Test Kit Using the Polysaccharide as a Genus-Specific Diagnostic Antigen for Leptospirosis in Korea, Bulgaria, and Argentina**

Sample selection

This is a retrospective study aimed to evaluate the accuracy of lateral flow immunoassays using archived serum samples collected in Korea, Bulgaria, and Argentina. There were 2 groups of samples selected for the study.

1. **Leptospirosis serum sample group**

For samples from Korea, 33 specimens from patients showing at least two leptospirosis-like symptoms (fever, chills, headache, muscle aches, vomiting, diarrhea, jaundice, pulmonary hemorrhage, or renal failure) were used. Every sample in these group were tested positive for leptospirosis by MAT.

For samples from Bulgaria, 25 specimens were selected from serum samples submitted to National Reference Vector-borne Infections Laboratory. Every patient had at least two symptoms of typical leptospirosis and in every case selected for the study were tested positive for leptospirosis by MAT.

For samples from Argentina, 100 leptospirosis patients in Argentina were randomly selected from the Instituto Nacional de Enfermedades Respiratorias. Each case was considered clinically compatible with and tested positive for leptospirosis by MAT.

All samples used in this evaluation were mostly taken in the acute phase of illness.

1. **Non-leptospirosis sample group**

In Korea, the serum samples from healthy donors (n = 23) and patients with acute febrile diseases other than acute febrile diseases leptospirosis, including scrub typhus (n = 25), hemorrhagic fever with renal syndrome (n = 21), and murine typhus (n = 25), were used in the study. These patients were confirmed negative for leptospirosis by MAT.

In Bulgaria, 25 control specimens were collected from five other febrile diseases which included rheumatoid arthritis (n = 5), EBV infectious mononucleosis (n = 5), multiple sclerosis (n = 5), Lyme disease (n = 5), and Syphilis (n = 5).

In Argentina, 108 specimens with healthy controls (n = 85) and three other acute febrile diseases, including dengue fever (n = 13), Argentina hemorrhagic fever (n = 5), and hemorrhagic fever with renal syndrome (n = 5), were used.

Reference tests

In this study, leptospirosis status of the leptospirosis serum samples was confirmed by **MAT**. However, the criteria were set differently for the different countries.

In Korea, all the sera classified as leptospirosis samples in the study had a single serum titer of ≥ 1:80 OR a ≥ four-fold rise in pair serum. Leptospiral serovars used in MAT panel included lai and canocola.

In Bulgaria, all the sera classified as leptospirosis samples in the study had a single serum titer of ≥ 1:200. Leptospiral serovars used in MAT panel included icterohaemorrhagiae, pomona, and bratislava.

In Argentina, all the sera classified as leptospirosis samples in the study had a single serum titer of ≥ 1:800 OR a ≥ four-fold rise in pair serum. Leptospiral serovars used in MAT panel included castellonis, canicola, grippothyphosa, copenhageni, icterohaemorrhagiae, pomona, pyrogenes, tarassovi, wolffi, hardjo, bataviae, patoc, australis, autumnalis, cynopteri, hebdomadis, javanica, panama, and sejroe.

Index tests

The index test evaluated in this study was **ImmuneMed Leptospira Rapid Test (ImmuneMed, South Korea).** The test was designed for detection of IgM and IgG against *Leptospira* (possibly polysaccharide antigen of nonpathogenic *Leptospira patoc*). All the cases were taken for evaluation in double-blind tests at each site.

Results

|  | **LEPkit** | | |
| --- | --- | --- | --- |
|  | **Korea** | **Bulgaria** | **Argentina** |
| True positive | 31 | 25 | 81 |
| False positive | 2 | 0 | 5 |
| True negative | 92 | 25 | 103 |
| False negative | 2 | 0 | 19 |
| total | 127 | 50 | 208 |
| **Sensitivity** | **93.9**  **(80.4-98.3** | **100**  **(84.3-100)** | **81.0**  **(72.2-87.5)** |
| **Specificity** | **97.9**  **(92.6-99.4)** | **100**  **(84.3-100)** | **95.4**  **(89.6-98.0)** |

**Eugene, 2015**

**﻿** **Evaluation of two immunodiagnostic tests for early rapid diagnosis of leptospirosis in Sri Lanka: a preliminary study**

Patient recruitment

This is a prospective study conducted in Sri Lanka between June to September 2010. Patients who were admitted to the hospitals with suspected leptospirosis symptoms were enrolled to the study. The following criteria, based on World Health Organisation-Leptospirosis Epidemiology Research Group (WHO-LERG) Epidemiological criteria, were used to define a leptospirosis case: acute febrile illness with the following: headache, myalgia, arthralgia, conjunctival suffusion, meningeal irritation, anuria, oliguria, proteinuria, jaundice, hemorrhages, cardiac arrhythmia, skin rash); or with a contact history of exposure to water or soil contaminated with urine of infected animals. After enrollment, serum sample were collected. There were 84 patients presenting with clinically suspected acute leptospirosis enrolled in this study.

Reference tests

In this study, **MAT** were used to define leptospirosis status of all 84 patients. Sera were considered positive for leptospirosis if MAT results showed a single serum titer of ≥ 1:400.

Serovar of *Leptospira* spp. used in MAT was *Leptospira biflexa* serovar Patoc strain Patoc-1.

Of 84 patients, the results showed that 40 patients were defined leptospirosis cases.

Index tests

The index test evaluated in this study was **Leptocheck WB** (Zephyr Biomedicals, India). The test was designed for detection of IgM against *Leptospira*. The evaluation was performed as described in the manufacturer’s manual.

Results

|  | **Leptocheck WB** | **Bayesian latent class model** |
| --- | --- | --- |
| True positive | 34 |  |
| False positive | 12 |  |
| True negative | 32 |  |
| False negative | 6 |  |
| total | 84 |  |
| **Sensitivity** | **85.0 (70.1 – 94.3)** | **95.0 (79.3 – 100)** |
| **Specificity** | **72.7 (57.2 – 85.0)** | **76.4 (60.8 – 93.2)** |

**Podgoršek, 2015**

**﻿** **Evaluation of the immunochromatographic (Leptocheck) test for detection of specific antibodies against leptospires**

Patient recruitment

This is a prospective study conducted in Slovenia. Patients who were visited the hospital with clinical manifestations suggestive for leptospirosis were enrolled to study. Patients were enrolled based on clinicians’ decision on patient’s clinical presentation and medical history. Blood samples were collected at the first patient’s visit to the hospital. For some patients, additional serum samples two or more weeks after first testing were available. There were 590 patients presenting with clinically suspected acute leptospirosis enrolled in this study.

Reference tests

In this study, **MAT, PCR and culture** were used to define leptospirosis status of the leptospirosis reference samples. Samples were defined as confirmed leptospirosis if:

1. **MAT** assay result showed:
2. a single serum titer of ≥ 1:100 OR
3. a seroconversion (undetectable acute-phase tier and convalescent phase titer ≥ 1:100) OR
4. **PCR** targeting *rrs* gene yielded a positive result OR
5. **Culture** showed the growth of leptospires

MAT panel included 13 leptospiral serovars: Gryppotyphosa, Canicola, Sejroe, Pomona, Cynopteri, Copenhageni, Patoc, Australis, Autumnalis, Pyrogenes, Bataviae, Panama, and Javanica.

Of 590 patients, the results showed that 35 patients were defined leptospirosis cases.

Index tests

The index test evaluated in this study was **Leptocheck WB** (Zephyr Biomedicals, India). The test was designed for detection of IgM against *Leptospira*. The evaluation was performed as described in the manufacturer’s manual. Only results from acute samples were used for the evaluation.

Results

|  | **Leptocheck WB** |
| --- | --- |
| True positive | 28 |
| False positive | 8 |
| True negative | 547 |
| False negative | 7 |
| total | 590 |
| **Sensitivity** | **80.0** |
| **Specificity** | **98.6** |

**Niloofa, 2015**

**﻿** **Diagnosis of Leptospirosis: Comparison between Microscopic Agglutination Test, IgM-ELISA and IgM Rapid Immunochromatography Test**

Patient recruitment

This is a prospective study conducted in Sri Lanka between June 2012 to May 2014. Patients over the age of 12 years with a suspected diagnosis of Leptospirosis and admitted to the hospitals were enrolled. A suspected diagnosis of leptospirosis was defined based on the World Health Organisation-Leptospirosis Epidemiology Research Group (WHO-LERG) Epidemiological criteria i.e., acute febrile illness with headache, myalgia, arthralgia, conjunctival suffusion, meningeal irritation, anuria, oliguria, proteinuria, jaundice, hemorrhages, cardiac arrhythmia or skin rash, or with a contact history of exposure to water or soil contaminated with urine of infected animals. Patients with a definitive alternative diagnosis on presentation, such as dengue, pneumonia, meningitis, or other bacterial sepsis, and pregnant women were excluded. After enrollment, serum sample were collected for all patients. Samples were collected at median of 6 days (SD±3.58) after the onset of symptoms. All enrolled patients who were available were requested to return for convalescent sampling on day 21 from disease onset. There was a total of 888 patients included in this study. Follow-up samples were received from 255/888 patients.

Reference tests

In this study, **MAT** was used to define leptospirosis status of the 888 patients. Patients were considered positive for leptospirosis if MAT results showed:

1. a single serum titer of ≥ 1:400 OR
2. a seroconversion (undetectable acute-phase tier and convalescent phase titer ≥ 1:100) OR
3. a ≥ four-fold rise in paired serum

Serovar of *Leptospira* spp. used in MAT was *Leptospira biflexa* serovar Patoc strain Patoc-1.

Among all these 888 patients, the MAT results showed that 354 patients were confirmed leptospirosis. Of these, 293 patients had a single MAT positive, and another 61 patients were positive based on paired MAT.

Index tests

The index test evaluated in this study was **Leptocheck WB** (Zephyr Biomedicals, India). The test was designed for detection of IgM against *Leptospira*. The evaluation was performed as described in the manufacturer’s manual. Only acute samples were tested.

Results

|  | **Leptocheck WB** | **Bayesian latent class model** |
| --- | --- | --- |
| True positive | 286 |  |
| False positive | 121 |  |
| True negative | 405 |  |
| False negative | 76 |  |
| total | 888 |  |
| **Sensitivity** | **80.8 (76.2–84.7)** | **86.2 (81.5–90.0)** |
| **Specificity** | **76.9 (73.0–80.4)** | **84.3 (80.3–87.7)** |

**Colt, 2014**

**﻿** **Human leptospirosis in The Federated States of Micronesia: a hospital-based febrile illness survey**

Patient recruitment

This is a prospective study conducted in The Federated States of Micronesia between June to September 2011. Eligible participants included individuals older than 10 years who presented to hospitals with reported fever or measured temperature ≥ 38 °C, and two or more of the following symptoms: headache, fatigue, myalgia, chills, conjunctival suffusion, anuria or oliguria, cough, jaundice, haemorrhages, vomiting, diarrhoea, meningeal irritation, cardiac arrhythmias, or skin rash. Exclusion criteria included diagnosed upper respiratory illness, pneumonia, dengue, or influenza. On average, patients presented to hospital 3 days (2.12-3.85 CI) following the onset of symptoms. Paired blood specimens were collected from the enrolled patients. A primary blood specimen was collected at enrolment, and a secondary blood specimen was collected during a follow-up study visit, between 10–30 days following the date of symptoms onset. During the study period, 54 participants enrolled were tested for leptospirosis by MAT. Of these, 29 patients received MAT test on both acute and convalescent samples.

Reference tests

In this study, **MAT** was used to define leptospirosis status of 83 serum samples collected. Sera were considered positive for leptospirosis if MAT results showed:

1. a single serum titer of ≥ 1:400 OR
2. a ≥ four-fold rise in paired serum

MAT panel included 21 leptospiral serovars: Pomona, Sejroe, Tarassovi, Grippotyphosa, Celledoni, Copenhageni, Australis, Canicola, Pyrogenes, Hebdomadis, Mini, Sarmin, Autumnalis, Cynopteri, Ballum, Bataviae, Djasiman, Javanica, Panama, Shermani and Pohnpei (LT751).

Among all these 54 patients (83 serum samples), the MAT results showed that 11 patients (13 serum samples) were confirmed leptospirosis.

Index tests

The index test evaluated in this study was **SD bioline Leptospira IgG/IgM** (Standard Diagnostics, South Korea). The test was designed for detection of IgM and IgG against *Leptospira interrogans*. The evaluation was performed as described in the manufacturer’s manual. All 83 serum samples were tested.

Results

|  | **SD bioline Leptospira IgG/IgM** |
| --- | --- |
| True positive | 9 |
| False positive | 7 |
| True negative | 63 |
| False negative | 4 |
| total | 83 |
| **Sensitivity** | **69.2 (42.3-89.3)** |
| **Specificity** | **90.0 (81.6-95.6)** |

**Chang, 2014**

**﻿** **﻿** **Limited diagnostic value of two commercial rapid tests for acute leptospirosis detection in Malaysia**

Sample selection

This is a retrospective study aimed to evaluate the accuracy of lateral flow immunoassays using archived serum samples collected in Malaysia in year 2012. They were samples that were sent to Institute for Medical Research (IMR), Kuala Lumpur, Malaysia for leptospirosis diagnosis based on the history and clinical presentations. These archived samples served as leptospirosis-confirmed samples and were divided into 2 groups based on the results of reference tests.

1. **Leptospirosis serum sample group I**

This group comprised of 58 serum samples that were tested positive for leptospirosis by MAT and PCR. Thus, this group represented acute phase samples at which *Leptospira* spp. in blood could be detected by PCR.

1. **Leptospirosis serum sample group II**

This group comprised of 55 serum samples collected from leptospirosis cases (MAT positive) whose blood samples were not available for PCR analysis. Thus, samples in this group could be either acute or convalescent samples.

1. **Control sample group**

A total of 70 samples (29 from healthy individuals and 41 from control patients) were also used to evaluate the accuracy of the index tests. Patient controls comprised those with dengue (n = 12); syphilis (n = 10); pyogenic liver abscess (n = 4); and parasitic infections such as malaria (n = 5), amoebiasis (n = 5), and toxoplasmosis (n = 5). All serum samples were from previously stored and anonymized samples.

Reference tests

In this study, **MAT and PCR** were used to confirm leptospirosis status of all 113 leptospirosis serum samples. Sera were considered positive for leptospirosis if:

1. **MAT** results showed a single serum titer of ≥ 1:400 AND
2. **PCR** targeting *Lepstospira secY* gene yielded a positive result (for group I samples only)

MAT panel included 18 leptospiral serovars: Patoc*,* Australis, Autumnalis, Ballum, Bataviae, Canicola, Celledoni, Hebdomadis, Cynopteri, Grippotyphosa, Icterohaemorrhagiae, Javanica, Pomona, Pyrogenes, Tarassovi, Hardjo, Sejroe, and Djasiman.

Index tests

The index test evaluated in this study was **VISITECT Lepto** (Omega Diagnostics Group PLC, UK). The test was designed for detection of IgM against Leptospiral antigen. Only 1 person performed the test to reduce subjectivity in test interpretation. If the test line looked faint and the technician doubted whether there was a visible line, the test was repeated. If the same result was observed, it was recorded as inconclusive. However, the inconclusive results were considered as positives for the data analysis.

Results

|  | **VISITECT Lepto** | |
| --- | --- | --- |
|  | **Group I sample (acute)** | **Group II sample (mixed)** |
| True positive | 14 | 22 |
| False positive | 4 | 4 |
| True negative | 66 | 66 |
| False negative | 44 | 33 |
| total | 128 | 125 |
| **Sensitivity** | **24** | **40** |
| **Specificity** | **94** | **94** |

**Widiyanti, 2013**

**﻿** **﻿** **Development of Immunochromatography-Based Methods for Detection of Leptospiral Lipopolysaccharide Antigen in Urine**

Patient recruitment

In this study, immunochromatography-based lateral ﬂow assays (LFA) for the diagnosis of leptospirosis was developed. A prospective study was conducted to evaluate the accuracy of the LFA. The study was conducted in Philippines. Serum and urine samples were collected from 44 patients with suspected leptospirosis and 14 healthy individuals. For all leptospirosis-suspected samples, they were collected 1 – 68 days after onset of illness.

Reference tests

In this study, **MAT and PCR** were used to confirm leptospirosis status of all 58 serum samples. Individuals were considered positive for leptospirosis if:

1. **MAT** results showed a single serum titer of ≥ 1:400 OR
2. **PCR** targeting *Lepstospira flaB* gene or *rrl* gene yielded a positive result

MAT panel included at least the following serovars: Copenhageni, Patoc, Poi, Manilae, Pyrogenes, Canicola, Semaranga, Losbanos, and Ratnapura. The full list of MAT panel used was not provided.

Index tests

The index test evaluated in this study was **immunochromatography-based lateral ﬂow assays (LFA)** and **dipstick** assay developed in-house. Both tests were designed for detection of LPS of *L. interrogans* in urine samples using mAb 1H6 that is specific to LPS of *L. interrogans* serovar Hebdomadis. The difference between LFA and dipstick developed in this study was a sample application step. A dipstick was designed to work with 96-well plate where urine samples and 1H6-gold conjugate were premixed while LFA was designed for samples to be dropped directly on the sample pad.

Results

|  | **immunochromatography-based lateral ﬂow assays (LFA)** | **Dipsticks** |
| --- | --- | --- |
| True positive | 31 | 28 |
| False positive | 3 | 6 |
| True negative | 20 | 17 |
| False negative | 4 | 4 |
| total | 58 | 58 |
| **Sensitivity** | **89 (78-96)** | **80 (70-90)** |
| **Specificity** | **87 (80-96)** | **74 (63-85)** |

**Goris, 2013**

**﻿** **﻿** **Prospective Evaluation of Three Rapid Diagnostic Tests for Diagnosis of Human Leptospirosis**

Patient recruitment

This is a prospective study conducted in The Netherlands between July 2001 to August 2012. During this period, all human blood specimens sent by physicians practicing in the Netherlands to NRL for leptospirosis diagnosis were tested upon arrival by routine diagnostics. In most cases only one sample was received per participant, in other cases two or more samples. Samples that were not tested by MAT or ELISA or not sufficient for RDT were excluded. As a result, a total of 5,144 samples were available for the study. Of these, 367 samples were confirmed leptospirosis. Samples collected included both acute and convalescent sera.

Reference tests

In this study, **MAT and IgM-ELISA** were used to confirm leptospirosis status of all 5,144 serum samples. Individuals were considered positive for leptospirosis if:

1. **MAT** assay result showed:
2. a single serum titer of ≥ 1:160 OR
3. a seroconversion/ ≥ four-fold rise in paired sera taken 2 days apart OR
4. **IgM-ELISA** assay result showed:
   1. a single serum titer of ≥ 1:160 OR
   2. a seroconversion/ ≥ four-fold rise in paired sera taken 2 days apart OR
5. **Culture** showed the growth of leptospires

MAT panel included 15 serovars: Bratislava, Ballum, Canicola, Grippotyphosa, Hebdomadis, Icterohaemorrhagiae, Copenhageni, Poi, Pomona, Proechimys, Hardjo, Saxkoebing, Sejroe and Patoc. An additional panel of 12 serovars was also tested for samples from participants who visited a country outside the Netherlands within one month prior to the day of onset of symptoms. This additional panel included Australis, Rachmati, Bataviae, Celledoni, Cynopteri, Mini, Panama, Pyrogenes, Shermani, Tarassovi, Andamana and Semaranga.

IgM-ELISA was performed from leptospires serovars Copenhageni.

Index tests

Two commercial lateral flow immunoassays (LFI) were evaluated:

1. **LeptoTex Lateral Flow** (Organon Teknika B.V. Boxtel, the Netherlands)

This assay was designed to detect IgM specific to *Leptospira*. The evaluation was performed using sampled collected during 2001-2004. A total number of samples used for evaluation of this test was 1,404 samples. Of these, 109 were positive for leptospirosis.

1. **Leptocheck WB** (Zephyr Biomedicals, India)

This assay was designed to detect IgM specific to *Leptospira*. The evaluation was performed using sampled collected during 2004-2012. A total number of samples used for evaluation of this test was 2,757 samples. Of these, 197 were positive for leptospirosis.

The tests were performed as described in the manufacturer’s manual.

Results

|  | **LeptoTex Lateral Flow** | | **Leptocheck WB** | |
| --- | --- | --- | --- | --- |
|  | **Acute** | **Mixed** | **Acute** | **Mixed** |
| True positive | 74 | 85 | 100 | 153 |
| False positive | 57 | 66 | 56 | 63 |
| True negative | 1235 | 1229 | 2495 | 2497 |
| False negative | 34 | 24 | 83 | 44 |
| total | 1,400 | 1,404 | 2,734 | 2,757 |
| **Sensitivity** | **69 (59-77)** | **78 (69-85)** | **55 (47-62)** | **78 (71-83)** |
| **Specificity** | **96 (94-97)** | **95 (94-96)** | **98 (97-98)** | **98 (97-98)** |

**Nabity, 2012**

**﻿** **﻿** **Accuracy of a Dual Path Platform (DPP) Assay for the Rapid Point-of-Care Diagnosis of Human Leptospirosis**

Sample selection

This is a retrospective study aimed to evaluate the accuracy of lateral flow immunoassays using archived serum samples collected in Salvador and Recife, Brazil. They were samples that were sent to Institute for Medical Research (IMR), Kuala Lumpur, Malaysia for leptospirosis diagnosis based on the history and clinical presentations. These archived samples served as leptospirosis-confirmed samples and were divided into 2 groups based on the results of reference tests.

1. **Leptospirosis serum sample group**

Samples in this group were selected from 3 different serum banks. First, 259 acute and 110 convalescent samples were selected from a serum bank of hospitalized case-patients ≥5 years of age with confirmed leptospirosis. The bank was created at the state reference infectious disease hospital in Salvador between 1996-2010. The inclusion criteria were i) strong clinical suspicion for leptospirosis or ii) at least one of the following: acute undifferentiated fever associated with either bleeding, acute renal insufficiency, jaundice, or acute liver injury with transaminases,1,000 U/L. Second, 23 acute samples were derived from a teaching hospital in Recife, Brazil between June–August 2010 using the same active surveillance inclusion criteria. Third, 28 acute and 26 convalescent samples were collected during community-based sentinel surveillance for acute febrile illness in Salvador between 2009-2010. All samples selected were tested and confirmed leptospirosis by MAT.

1. **Control serum sample group**

Control group of sera comprised of healthy control sera and sera from non-leptospirosis patients. Healthy control sera were derived from: 1) Salvador slum residents (n=162), 2) Salvador blood donors (n=150), and 3) U.S. blood donors (n=100). Non-leptospirosis patient sera included i) dengue infection (n=65), hepatitis A infection (n=65), syphilis (n=65), acute febrile illness (n=70). All this control sera were tested negative for leptospirosis.

Reference tests

In this study, **MAT** was used to confirm leptospirosis status of 1,123 leptospirosis serum sample. Sera were considered positive for leptospirosis if MAT results showed:

1. a single serum titer of ≥ 1:800 OR
2. a ≥ four-fold rise in paired serum
3. a seroconversion (undetectable acute titer and convalescent titer ≥1:200)

MAT panel included 25 leptospiral serovars: Copenhageni, Canicola, Patoc, Autumnalis, Ballum, Grippotyphosa, Cynopteri, Shermani, Tarassovi, Bratislava, Castellonis, Bataviae, Celledoni, Djasiman, Hebdomadis, Icterohaemorrhagiae, Coxi, Louisiana, Panama, Pomona, Pyrogenes, Hardjo, Wolffi, Canalzonae, and Mini.

Index tests

The index test evaluated in this study was **Dual Path Platform** (DPP, Chembio Diagnostic Systems, USA). The test was designed for detection of antibody against recombinant leptospiral immunoglobulin-like (rLig) antigen. To evaluate the accuracy of the test, DPP assays were performed per manufacturer instruction using 3 independent operators visually interpreted results after 20 minutes. Interpreters were blinded to case patient status.

Results (reported)

| **Case group** | | | | **Control group** | | | |
| --- | --- | --- | --- | --- | --- | --- | --- |
|  | N | % sen | DPP (+) |  | N | % spec | DPP (-) |
| Severe, Salvador, acute | 259 | 85 | 220 | Healthy slum residents | 162 | 86 | 139 |
| Severe, Salvador, convalescent | 110 | 98 | 108 | Healthy donor Brazil | 150 | 93 | 140 |
| Severe, Recife, acute | 23 | 78 | 18 | Healthy donor US | 100 | 98 | 98 |
| Mild, Salvador, acute | 28 | 64 | 18 | Dengue cases | 65 | 100 | 65 |
| Mild, Salvador, convalescent | 26 | 50 | 13 | Hepatitis A cases | 65 | 95 | 62 |
| **total** | **446** |  |  | Syphilis cases | 65 | 95 | 62 |
|  |  |  |  | Acute febrile cases | 70 | 99 | 69 |
|  |  |  |  | **total** | **677** |  |  |

Results (recalculated)

|  | **Dual Path Platform** | |
| --- | --- | --- |
|  | **Acute** | **convalescent** |
| True positive | 256 | 121 |
| False positive | 42 | 42 |
| True negative | 635 | 635 |
| False negative | 54 | 15 |
| total | 987 | 813 |
| **Sensitivity** | **82.6** | **89.0** |
| **Specificity** | **93.8** | **93.8** |

**Silpasakorn, 2011**

**﻿** **﻿** **Performance of Leptospira Immunoglobulin M ELISA and Rapid Immunoglobulin G Immunochromatographic Assays for the Diagnosis of Leptospirosis**

Sample selection

This study aimed to evaluate the accuracy of lateral flow immunoassays using archived serum samples collected in Thailand. There were 2 groups of serum samples used in this study.

1. **Leptospirosis serum sample group**

The samples in this group were selected from stored, characterized sera collected from febrile patients in Thailand. Serum samples from the acute and convalescence phase of patients with leptospirosis were collected 2-45 days after the onset of fever from 89 patients (74 males and 15 females), aged 15- 84 years (mean age 40.5 years).

1. **Control serum sample group**

Control serum samples were obtained from 72 patients with the following diagnosis: dengue infection (17), scrub typhus (10), murine typhus (10), malaria (6), influenza A and influenza B (17), R. Helvetica infection (5), melioidosis and septicemia from other bacteria (7). None of the samples from this group reacted to MAT at serum titers ≥ 1: 100.

Reference tests

In this study, **MAT and Culture** were used to diagnose leptospirosis of all 161 serum samples. Samples were considered positive for leptospirosis if:

1. **MAT** assay result showed a ≥ four-fold rise in paired sera OR
2. **Blood** **culture** showed the growth of leptospires

List of serovar used in MAT was not reported.

Index tests

The index test evaluated in this study was **SD bioline leptospirosis** (Standard Diagnostics, South Korea). The test was designed for detection of IgG against *Leptospira interrogans*. Evaluation was performed per manufacturer instruction using 1 reader visually interpreted results after 15 minutes. The interpreter was blinded to patient status.

Results

|  | **SD bioline leptospirosis** |
| --- | --- |
| True positive | 74 |
| False positive | 1 |
| True negative | 71 |
| False negative | 15 |
| total | 161 |
| **Sensitivity** | **83.2** |
| **Specificity** | **98.6** |

**Cohen, 2006**

**Rapid diagnostic tests for dengue and leptospirosis: antibody detection is insensitive at presentation**

Patient recruitment

This is a prospective study conducted in Thailand between February 2002 to February 2003. Patients with the age of 6 years or older who presented to hospitals with a documented fever of > 38.0 °C were enrolled to the study. Patients were not eligible for the study if they had fever lasting longer than 2 weeks, an infection with a pathogen indisputably identiﬁable on clinical grounds (e.g. chickenpox), an immunization in the preceding 48 h, receipt of blood or blood products (e.g. plasma or IVIG) in the previous 6 months, or illness severe enough to require immediate transfer to a regional hospital. Patients were asked to return to the hospital 3–5 weeks after their initial visit for a repeat set of diagnostic tests. As a result, a total of 723 subjects were enrolled for the study. Convalescent sera were obtained from 704 patients. Of the patients with convalescent visits, 67 had confirmed leptospirosis.

Reference tests

In this study, **MAT** was used to define leptospirosis status of all patients. Individuals were considered positive for leptospirosis if MAT assay result showed a ≥ four-fold rise between acute and convalescent sera.

MAT tests were performed using 23 reference strains of *L. interrogans*. A list of MAT panel, however, was not reported.

Index tests

The index test evaluated in this study was **Multi-Test Dip-S-Ticks, DSLST** (PanBio Ltd. Australia and PanBio Inc. USA). The test was designed for detection of IgM against 5 pathogens including *L. interrogans* and *L. biflexa*.

Results

|  | **Multi-Test Dip-S-Ticks, DSLST**  **(convalescent)** |
| --- | --- |
| True positive | 55 |
| False positive | 121 |
| True negative | 516 |
| False negative | 12 |
| total | 706 |
| **Sensitivity** | **82** |
| **Specificity** | **81** |

**Blacksell, 2006**

**Limited Diagnostic Capacities of Two Commercial Assays for the Detection of Leptospira Immunoglobulin M Antibodies in Laos**

Patient recruitment

This is a prospective study conducted in Loas between November 2001 to October 2003. Human sera were collected as part of a study to determine the causes of unexplained fever for patients presenting at Mahosot Hospital, Laos. Paired admission and convalescent-phase serum specimens were available from 186 patients (total sample, n=372). The median interval between admission and convalescent-phase serum collection was 4.5 days (IQR 2 to 8). Unpaired sera were not included in the study.

Reference tests

In this study, **MAT** was used to define leptospirosis status of all patients enrolled. Individuals were considered positive for leptospirosis if MAT assay result showed:

1. a single serum titer of ≥ 1:400 OR
2. a ≥ four-fold rise in paired serum

MAT tests were performed at WHO/FAO/OIE Collaborating Centre for Reference and Research on Leptospirosis in The Netherlands and Australia. The Australian panel of serovars included Pomona, Hardjo, Tarassovi, Grippotyphosa, Celledoni, Copenhageni, Australis, Pyrogenes, Canicola, Hebdomadis, Mini, Sarmin, Autumnalis, Cynopteri, Ballum, Bataviae, Djasiman, Javanica, Panama, Shermani, and Mwalok. The Netherlands panel comprised of 26 serovars of leptospires. Additional serovars included Bratislava, Icterohaemorrhagiae, Poi, Proechimys, Saxkoebing, Sejroe, Patoc, Andamana, Rachmati, and Semaranga.

Among 186 patients enrolled, 23 were true leptospirosis (as defined by MAT).

Index tests

The index test evaluated in this study was **Leptotek** (Organon-Teknika, Netherlands). The test was designed for detection of IgM against *Leptospira*. Evaluation was performed according to the manufacturer’s instructions. All results were read by eye by the same operator.

Results

|  | **Leptotek** | |
| --- | --- | --- |
| Sample collection | **Admission**  **days 9 (7-14) of fever** | **Acute**  **days 6 (5-7) of fever** |
| True positive | 11 | 7 |
| False positive | 40 | 15 |
| True negative | 123 | 45 |
| False negative | 12 | 3 |
| total | 186 | 70 |
| **Sensitivity** | **47.3 (26.8–69.4)** | **70.0 (34.8–93.3)** |
| **Specificity** | **75.5 (68.1–81.9)** | **75.0 (62.1–85.3)** |

**Sehgal, 2003**

**Field application of Lepto lateral ﬂow for rapid diagnosis of leptospirosis**

Patient recruitment

This is a prospective study conducted in South Andaman between October 1999 to December 2000. Patients attending the outpatient department of a Primary Health Centre (PHC) with clinical suspicion of leptospirosis were enrolled to the study. The criteria for suspecting leptospirosis were the presence of fever, headache and body aches associated with any of the following symptoms: (i) calf muscle tenderness, (ii) bleeding tendencies, including subconjunctival haemorrhage, (iii) cough, haemoptysis and breathlessness, (iv) jaundice and (v) oliguria. As a result, a total of 117 patients were included. Blood samples were collected from all these patients on the day of reporting. A second sample was collected during weeks 2–4 of the illness. Second samples could be collected from only 104 patients.

Reference tests

In this study, **MAT and culture** were used to confirm leptospirosis status of all serum samples. Individuals were considered positive for leptospirosis if:

1. **MAT** assay result showed:
2. a single serum titer of ≥ 1:400 OR
3. a seroconversion (from seronegative to a titer of ≥ 1:100) OR
4. a ≥ four-fold rise in paired sera OR
5. **Culture** showed the growth of leptospires

MAT panel included 12 serovars: Grippotyphosa, Australis, Ballum, Lai, Pyrogenes, Tarassovi, Pomona, Rachmati, Canicola, Poi, Hebdomadis and Hardjo.

Among 117 patients enrolled, 70 were defined as cases of leptospirosis.

Index tests

The index test evaluated in this study was **Lepto Lateral flow** (The Royal Tropical Institute, The Netherlands). The test was designed for detection of IgM antibody specific to heat-extracted antigen prepared from Patoc 1 strain. Evaluation was performed according to the recommended procedure. The test was done on the ﬁrst and second samples.

Results

|  | **Lepto Lateral flow** | |
| --- | --- | --- |
| Sample collection | **Acute**  **first week of illness** | **Convalescent**  **week 2-4 of illness** |
| True positive | 37 | 49 |
| False positive | 3 | 5 |
| True negative | 44 | 42 |
| False negative | 33 | 8 |
| total | 117 | 104 |
| **Sensitivity** | **52.9** | **86.0** |
| **Specificity** | **93.6** | **89.4** |
