# Supplementary material for: Accuracy of rapid lateral flow immunoassays for human leptospirosis diagnosis: A systematic review and meta-analysis
Source: PLoS Negl Trop Dis. 2024 May 15;18(5):e0012174. doi: 10.1371/journal.pntd.0012174 (PMC11132494; doi:10.1371/journal.pntd.0012174)
Supplement: Attachment — Submitted filename: Corrections LFI 02032024.docx [file pntd.0012174.s016.docx]

**Review**

Paper: Accuracy of rapid lateral flow immunoassays for human leptospirosis diagnosis: a systematic review and meta-analysis

**Observations**

| **Line** | **Observations** |
| --- | --- |
| 1 | The name registered in PROPERO for the register of the systematic review is different from the name used in this paper (In PROSPERO the authors use the word “Performance”, here they use the word “Accuracy”) |
| 63 | Clarify your idea, transmission occurs through direct contact with fluids from infected animals, not the animal itself. |
| 65-64 | Fever might be the most common symptom among patients who seek medical care, but most patients present asymptomatic of interspecific symptoms. |
| 73 | Replace “in the genus of” by belonging to the genus |
| 108 | IFA is not considered a reference test for the diagnosis of leptospirosis, only MAT, culture, or PCR. However, it can be taken into account because this test generates a quantitative result of 1:400 or a fourfold increase in the control sample. The authors made reference to the reason they included studies that used IFA as a reference test in the discussion (lines 304-306), however, they might consider making this clear in the methodology. |
| 159 | Review the grammar of that sentence. (remove “for reasons”) |
| 173 | Review the grammar of that sentence. (remove the s from “numbers”, remove the “were”, and remove the “per study” (the variation in sample sizes was among the different studies not within each study)) |
| 184-192 | At the start of the paragraph when you cite the number of studies you include the percentage they represent in your sample (lines 184 and 185) however after that you just cite the number of studies without providing the percentage they represent (lines 185, 187, 188, and 192). Use one or the other but try to keep the same format. |
| 379 | 20 represents the number of LFIs used by the different studies included in your findings. However, can you state this corresponds to the number of developed and marketed LFIs (remember that you only included studies in English)? |

Methods
-Are the objectives of the study clearly articulated with a clear testable hypothesis stated?
-Is the study design appropriate to address the stated objectives?
-Is the population clearly described and appropriate for the hypothesis being tested?
-Is the sample size sufficient to ensure adequate power to address the hypothesis being tested?
-Were correct statistical analysis used to support conclusions?
-Are there concerns about ethical or regulatory requirements being met?

The objective is clear, and the study design is appropriate, authors were strict in adhering to guidelines and in the review of the studies. However, not including articles in Spanish and Portuguese (among other languages) might limit the results as regions that speak these languages produce a lot of research on leptospirosis.

Results
-Does the analysis presented match the analysis plan?
-Are the results clearly and completely presented?
-Are the figures (Tables, Images) of sufficient quality for clarity?

The results are clear and adequate for the type of study, figures, and tables are sufficient and clear, and well complimented with the supplements. However, in your Forest Plot analysis, you show an I^2^ value of 95.76 for sensitivity and 96.35 for specificity which suggests that the studies included might have been too heterogeneous for a metanalysis.

Conclusions
-Are the conclusions supported by the data presented?
-Are the limitations of analysis clearly described?
-Do the authors discuss how these data can be helpful to advance our understanding of the topic under study?
-Is public health relevance addressed?

There is no conclusion section, some conclusions are presented in the discussion but a section with the author's main conclusion presented in a clear and summarized way is needed.

Public health relevance of the study results is not well addressed, the authors could take a stand on whether they believe current evidence supports the use of LFIs in clinical practice (exp: useful, not useful, or not enough evidence to determine their usefulness).

Editorial and Data Presentation Modifications?
Use this section for editorial suggestions as well as relatively minor modifications of existing data that would enhance clarity. If the only modifications needed are minor and/or editorial, you may wish to recommend “Minor Revision” or “Accept”.

The study was clear and well done, the subject is important because once again. The findings point to the fact that rapid tests (LFIs) do not have an adequate performance for the diagnosis of leptospirosis and the reference tests (MAT, PCR, and culture) are difficult to implement and apply in areas where the disease prevails, which leads to underdiagnosis, under-registration and inadequate treatment.

I suggest it be accepted after minor modifications.

**Ethical Research and Reporting**

Has all research been conducted to a high ethical standard, and is the work performed the same work which was approved? (Note that PLOS NTDs staff runs an internal ethics check before papers proceed to review, so this is a secondary check.)

All research involving human participants must have been approved by the authors' Institutional Review Board or an equivalent committee, and all clinical investigation must have been conducted according to the principles expressed in the Declaration of Helsinki. Informed consent, written or oral, should also have been obtained from the participants. A brief overview of the ethics reporting should be provided in the Methods; if no consent was given, the reason must be explained.

This does not apply to the study in question because a systematic review and meta-analysis do not have direct study subjects and are considered studies No Risk studies, however, the authors could make reference that they considered the study a no risk study and no approval from ethical committee was needed
