# Supplementary material for: Accuracy of rapid lateral flow immunoassays for human leptospirosis diagnosis: A systematic review and meta-analysis
Source: PLoS Negl Trop Dis. 2024 May 15;18(5):e0012174. doi: 10.1371/journal.pntd.0012174 (PMC11132494; doi:10.1371/journal.pntd.0012174)
Supplement: Attachment — Submitted filename: PNTD-D-24-00158 Response to Reviewers.docx [file pntd.0012174.s017.docx]

**Response to Reviewers**

Dear Dr. Biswal and Dr. Nascimento,

Subject: Response to the reviews’ comments for the manuscript **PNTD-D-24-00158**

Thank you for the time and effort that you and the reviewers dedicated to review our manuscript entitled “**Accuracy of rapid lateral flow immunoassays for human leptospirosis diagnosis: a systematic review and meta-analysis**”. We are grateful for the opportunity to submit a revised manuscript for publication in the PLOS Neglected Tropical Diseases. Please see below for a point-by-point response to the reviewers’ comments and concerns. All the suggestions and concerns made by the reviewers have been considered and the changes are highlighted within the manuscript.

**METHODS**

**Reviewer #1:** The objectives of the study are clearly stated, and the study design is appropriate. Databases searched were described, but I miss the "keywords" and specific dates of the search. Selection criteria are there and correct statistical analyses were used to draw conclusions.

**Response:** Thank you for the valuable comments and notices. We agree that the specific date of the search is missing, and it will be added to the manuscript. The “keywords” of search, however, are shown in S1 Appendix.

**Action:** Line 100 has been modified to include the specific dates of the search.

**Reviewer #2:** The objective is clear, and the study design is appropriate, authors were strict in adhering to guidelines and in the review of the selected studies. However, not including articles in Spanish and Portuguese (among other languages) might limit the results as regions that speak these languages produce a lot of research on leptospirosis.

**Response:** Thank you for the suggestions. We agree that exclusion of articles published in other languages, especially Spanish and Portuguese, might limit the results. This concern will be added to the result section.

**Action:** We have added the sentences “However, it should be noted that, in this study, the articles published in non-English languages were excluded. Thus, the total number of 20 LFIs included in this review may not correspond to the number of all developed LFIs.” to the result section. See lines 180 – 182.

**RESULTS**

**Reviewer #1:** Results are clearly described and match with the methods. Results of the quality assessment are assessed and presented in good quality and understable way.

**Response:** Thank you for the comments

**Action:** None

**Reviewer #2:** The results are clear and adequate for the type of study, figures, and tables are sufficient and clear, and well complimented with the supplements. However, in your Forest Plot analysis, you show an I2 value of 95.76 for sensitivity and 96.35 for specificity which suggests that the studies included might have been too heterogeneous for a metanalysis.

**Response:** We agree with the reviewer’s observation that the studies included are very heterogeneous and perhaps too heterogeneous for a metanalysis as suggested by the I^2^ values. We have mentioned this issue already at lines 320 – 322 in the discussion section. The manuscript, however, will be modified to point out about the I^2^ values and to stress about the heterogeneity of the included studies.

**Action:** The sentence “The I^2^ values for sensitivity and specificity were 95.76% and 96.35%, respectively, indicating that the data entries included might be too heterogeneous to achieve accurate meta-analysis results.” has been added to the results section (see lines 251-253). In discussion, the sentence “Furthermore, the forest plot's I^2^ values indicate that the included studies were considerably heterogeneous to produce dependable meta-analysis results.” has been added (see lines 322-324).

**CONCLUSIONS**

**Reviewer #1:** Conclusions are supported by the data presented. It is not easy to draw conclusions on the sensitivity and specificity of leptospirosis diagnostic LFIs, when there is so much heterogeneity among the tests. This has been explained very well, together with limitations and considerations. Final conclusion in the last paragraph could be a bit more strong.

**Response:** As commented by the reviewer, we also think that it is challenging to determine whether the LFIs are clinically useful based on our findings. We will modify the conclusion section to include our opinion on the public health relevance of the study.

**Action:** The sentence “On the basis of our findings, it is difficult to ascertain whether the LFIs are clinically useful.” has been added to the manuscript (see lines 396-397).

**Reviewer #2:** There is no conclusion section, some conclusions are presented in the discussion but a section with the author's main conclusions presented in a clear and summarized way is needed.
Public health relevance of the study results is not well addressed, the authors could take a stand on whether they believe current evidence supports the use of LFIs in clinical practice (exp: useful, not useful, or not enough evidence to determine their usefulness).

**Response:** In our opinion, it is challenging to determine whether the LFIs are clinically useful based on our findings, and we recommend that further evaluations of LFIs are needed.

**Action:** The sentence “On the basis of our findings, it is difficult to ascertain whether the LFIs are clinically useful.” has been added to the manuscript (see lines 396-397).

**Summary and General Comments**

**Reviewer #1:** Significant, well-written study, that tries to fill a clinical gap about the accuracy of rapid diagnostic tests for human leptospirosis. Although the heterogenicity of the tests limits the authors to give some clinical relevant directions and novelty, the authors were able to formulate the answers on their objectives.

**Response:** Thank you for the compliments to our manuscript

**Action:** None

**Reviewer #2:** The study was clear and well done, the subject is important because once again. The findings point to the fact that rapid tests (LFIs) do not have an adequate performance for the diagnosis of leptospirosis and the reference tests (MAT, PCR, and culture) are difficult to implement and apply in areas where the disease prevails, which leads to underdiagnosis, under-registration and inadequate treatment.

**Response:** Thank you for your appreciation to our work

**Action:** None

**Editorial and Data Presentation Modifications**

**Reviewer #1**: No editorial comments.

**Response:** Thank you for your opinion

**Action:** None

**Reviewer #2**: I suggest it be accepted after minor modifications.

**Response:** Thank you for suggesting the manuscript to be accepted after minor revision

**Action:** The manuscript has been modified and corrected according to observations described below.

**OBSERVATIONS**

**Line 1:** The name registered in PROPERO for the register of the systematic review is different from the name used in this paper (In PROSPERO the authors use the word “Performance”, here they use the word “Accuracy”)

**Response:** Thank you for the notice. If it is possible, we would like to use the word “Accuracy” for the title of the paper.

**Action:** We have made a note in the PROSPERO protocol that the title is changed to “Accuracy of rapid lateral flow immunoassays for human leptospirosis diagnosis: a systematic review and meta-analysis”

**Line 63:** Clarify your idea, transmission occurs through direct contact with fluids from infected animals, not the animal itself.

**Response:** We agree that this statement should be clarified.

**Action:** The sentence has been changed to “Humans may acquire the infection from direct contact with infected animals’ fluids or from water or soil contaminated with those through cuts, abrasive skin, or mucosal contact” (see lines 63 – 65).

**Lines 65-64:** Fever might be the most common symptom among patients who seek medical care, but most patients present asymptomatic of interspecific symptoms.

**Response:** We agree to modify the texts according to the comment.

**Action:** Lines 65 – 67 have been changed to “Leptospirosis cases are usually asymptomatic, but some patients may develop symptoms. The most common symptom observed among leptospirosis patients is an acute febrile illness”.

**Line 73:** Replace “in the genus of” by belonging to the genus

**Response:** Thank you for the suggestion.

**Action:** The words “in the genus of” were replaced with “belonging to the genus” (see line 74).

**Line 108:** IFA is not considered a reference test for the diagnosis of leptospirosis, only MAT, culture, or PCR. However, it can be taken into account because this test generates a quantitative result of 1:400 or a fourfold increase in the control sample. The authors made reference to the reason they included studies that used IFA as a reference test in the discussion (lines 304-306), however, they might consider making this clear in the methodology.

**Response:** We agree to add those reasons in the methodology section.

**Action:** The reasons for including studies with IFA as a reference test have been added to the methodology section in the manuscript (see lines 112-113).

**Line 159:** Review the grammar of that sentence. (remove “for reasons”)

**Response:** Thank you for the suggestion.

**Action:** The words “for reasons” have been removed (see lines 161-163).

**Line 173:** Review the grammar of that sentence. (remove the s from “numbers”, remove the “were”, and remove the “per study” (the variation in sample sizes was among the different studies not within each study))

**Response:** Thank you for the suggestion.

**Action:** The sentence has been modified to “The number of patients or samples varied from 50 to approximately 2,800” (see line 175).

**Lines 184-192:** At the start of the paragraph when you cite the number of studies you include the percentage they represent in your sample (lines 184 and 185) however after that you just cite the number of studies without providing the percentage they represent (lines 185, 187, 188, and 192). Use one or the other but try to keep the same format.

**Response:** We agree that they should be in the same format throughout the paragraph.

**Action:** The percentages representing the numbers of studies cited have been added throughout the paragraph. See lines 189, 191, 193, and 196.

**Line 379:** 20 represents the number of LFIs used by the different studies included in your findings. However, can you state this corresponds to the number of developed and marketed LFIs (remember that you only included studies in English)?

**Response:** We agree that the number of developed and marketed LFIs could be higher than 20 if non-English articles were included. Thus, we will make a statement to emphasize this concern in the result section and modify line 387.

**Action:** We have changed the word “about” in line 381 to “at least”. Also, the statement regarding this concern has been added to the result section (see lines 180 – 182)

Sincerely,

Teerapat Nualnoi, Ph.D.

Lecturer

Department of Pharmaceutical Technology

Faculty of Pharmaceutical Sciences

Prince of Songkla University

teerapat.n@psu.ac.th
