# Supplementary material for: Accuracy of rapid lateral flow immunoassays for human leptospirosis diagnosis: A systematic review and meta-analysis
Source: PLoS Negl Trop Dis. 2024 May 15;18(5):e0012174. doi: 10.1371/journal.pntd.0012174 (PMC11132494; doi:10.1371/journal.pntd.0012174)
Supplement: Attachment — Submitted filename: PNTD-D-24-00158R1 Response to Reviewers.docx [file pntd.0012174.s018.docx]

**Response to Reviewers**

Dear Dr. Biswal and Dr. Nascimento,

Subject: Response to the reviews’ comments for the manuscript **PNTD-D-24-00158R1**

Thank you for the time and effort that you and the reviewers dedicated to review our manuscript entitled “**Accuracy of rapid lateral flow immunoassays for human leptospirosis diagnosis: a systematic review and meta-analysis**”. We are grateful for the opportunity to resubmit the manuscript for publication in the PLOS Neglected Tropical Diseases. Please see below for responses to the important additional instructions. Please note that there are no further reviewer comments to be addressed in this version of the manuscript.

**Figure files:**

We uploaded all the figures to the Preflight Analysis and Conversion Engine (PACE) digital diagnostic tool as instructed. The figures reuploaded to the submission system had been processed and verified by PACE already.

**Data Requirements:**

We would like to confirm that all relevant data are within the manuscript and its Supporting Information files.

**Reproducibility:**

We would like to confirm that protocols are described within the manuscript and registered with the International Prospective of Systematic Reviews (PROSPERO registration number CRD42022371788).

**References:**

We have reviewed the reference list and confirmed that it is complete and correct. There are no retracted papers cited in the manuscript.

Sincerely,

Teerapat Nualnoi, Ph.D.

Lecturer

Department of Pharmaceutical Technology

Faculty of Pharmaceutical Sciences

Prince of Songkla University

teerapat.n@psu.ac.th
